# The oral drug obeldesivir protects nonhuman primates against lethal Ebola virus infection

**DOI:** 10.1126/sciadv.adw0659

**Published:** 2025-03-14

**Authors:** Courtney Woolsey, Robert W. Cross, Victor C. Chu, Abhishek N. Prasad, Krystle N. Agans, Viktoriya Borisevich, Daniel J. Deer, Mack B. Harrison, Jasmine K. Martinez, Natalie S. Dobias, Karla A. Fenton, Tomas Cihlar, Anh-Quan Nguyen, Darius Babusis, Roy Bannister, Meghan S. Vermillion, Thomas W. Geisbert

**Affiliations:** ^1^ Galveston National Laboratory, University of Texas Medical Branch, Galveston, TX, USA.; ^2^ Department of Microbiology and Immunology, University of Texas Medical Branch, Galveston, TX, USA.; ^3^ Gilead Sciences Inc., Foster City, CA, USA.

## Abstract

Obeldesivir (ODV; GS-5245) is an orally administered ester prodrug of the parent nucleoside GS-441524 that has broad spectrum antiviral activity inhibiting viral RNA–dependent RNA polymerases. We recently showed that ODV completely protects cynomolgus macaques against lethal infection with Sudan virus when given 24 hours after parenteral exposure. Here, we report that once daily oral ODV treatment of cynomolgus and rhesus macaques for 10 days confers 80 and 100% protection, respectively, against lethal Ebola virus infection when treatment is initiated 24 hours after mucosal (conjunctival) exposure. ODV treatment delayed viral replication to abate excessive inflammation and promote adaptive immunity. For outbreak response, oral antivirals might present substantial advantages over now approved intravenous drugs, such as easy supply, storage, distribution, and administration. Furthermore, these results support the potential of ODV as an oral postexposure prophylaxis with broad spectrum activity across filoviruses.

## Teaser

Oral treatment with obeldesivir protects macaques from lethal infection with Ebola virus.

## INTRODUCTION

Viruses within the family *Filoviridae* cause high lethality in humans and nonhuman primates (NHPs), with mortality rates in some outbreaks approaching 90% (*1*, *2*). Filoviruses are classified as tier 1 select agents by the US Government as they present a risk of deliberate misuse with considerable potential for mass casualties or destructive effects to the economy, critical infrastructure, or public safety (*3*). Viruses within the *Orthoebolavirus* genus have caused the majority of filovirus outbreaks in endemic areas of Central and Western Africa. Among the four species of the genus *Orthoebolavirus*, viruses of the *Orthoebolavirus zairense* [Ebola virus (EBOV)] species have been the most notable threat to public health, with a number of major episodes over the past decade. The 2013–2016 West African epidemic of Ebola virus disease (EVD) caused 28,600 cases with 11,325 deaths (*4*), while the 2018–2020 EBOV outbreak in the Democratic Republic of Congo (DRC) and Uganda resulted in 3481 cases and 2299 deaths (*5*).

Since its discovery in 1976, efforts were made to develop vaccines and treatments for EVD. However, it was not until the 2013–2016 West African EBOV epidemic that substantial progress was made in advancing interventions for human use. From the time of this epidemic through the 2018–2020 EBOV outbreak in the DRC, two vaccines and two human monoclonal antibody (mAb)–based treatments were developed and licensed (*6*–*9*). While mAb-based treatments have shown success in preclinical animal models and in human clinical trials, their combination of cold-chain transport and storage requirements and intravenous route of administration pose substantial challenges in outbreak settings. Thus, there remains a need for EVD countermeasures that can be more rapidly and widely deployed (e.g., with oral agents) in resource-limited regions.

Obeldesivir (ODV) is a nucleoside analog prodrug with broad spectrum activity across several RNA virus families, including filoviruses. We have recently reported that ODV (100 mg/kg) protected cynomolgus macaques against a lethal challenge with *Orthoebolavirus sudanense* [prototype Sudan virus (SUDV)] when administered orally beginning 24 hours after a high dose SUDV parenteral exposure (*10*); moreover, ODV demonstrated potent in vitro antiviral activity against several filoviruses, including SUDV, EBOV, and Marburg virus (*10*). Here, we report the in vivo efficacy of ODV against lethal infection with the Makona variant of EBOV in both cynomolgus and rhesus macaques challenged by the mucosal exposure route.

Our previous ODV treatment study of SUDV-infected macaques evaluated efficacy in NHPs challenged with the conventional 1000 plaque-forming unit (PFU) dose of virus by the intramuscular route, which results in a more compressed disease course compared with more common natural human infections via mucosal exposure, making it difficult to fairly assess postexposure interventions across a relevant therapeutic window. For example, data from a clinical trial of several treatments in human EBOV-infected patients known as the PALM trial (abbreviated from “*pamoja tulinde maisha*,” Swahili for “together save lives”) (*11*) suggest that the interventions were not given until 10 to 12 days after exposure, whereas parenterally infected macaques succumb on average 6 to 8 days after EBOV challenge (*12*, *13*). Notably, in our laboratory, EBOV infection of macaques progresses faster than SUDV infection of macaques when animals are exposed by intramuscular injection (*13*), potentially affecting the efficacy of postexposure prophylaxis (PEP). Here, we assessed the ability of ODV to protect cynomolgus and rhesus macaques from lethal EVD when animals are exposed by a more natural mucosal route. We previously showed that mucosal exposure of cynomolgus monkeys to EBOV, while still being uniformly lethal, results in a more protracted disease course than parenteral exposure and better reflects the human condition (*14*).

## RESULTS

### Experimental challenge of cynomolgus macaques with EBOV and treatment with ODV

**Fig. 1.**
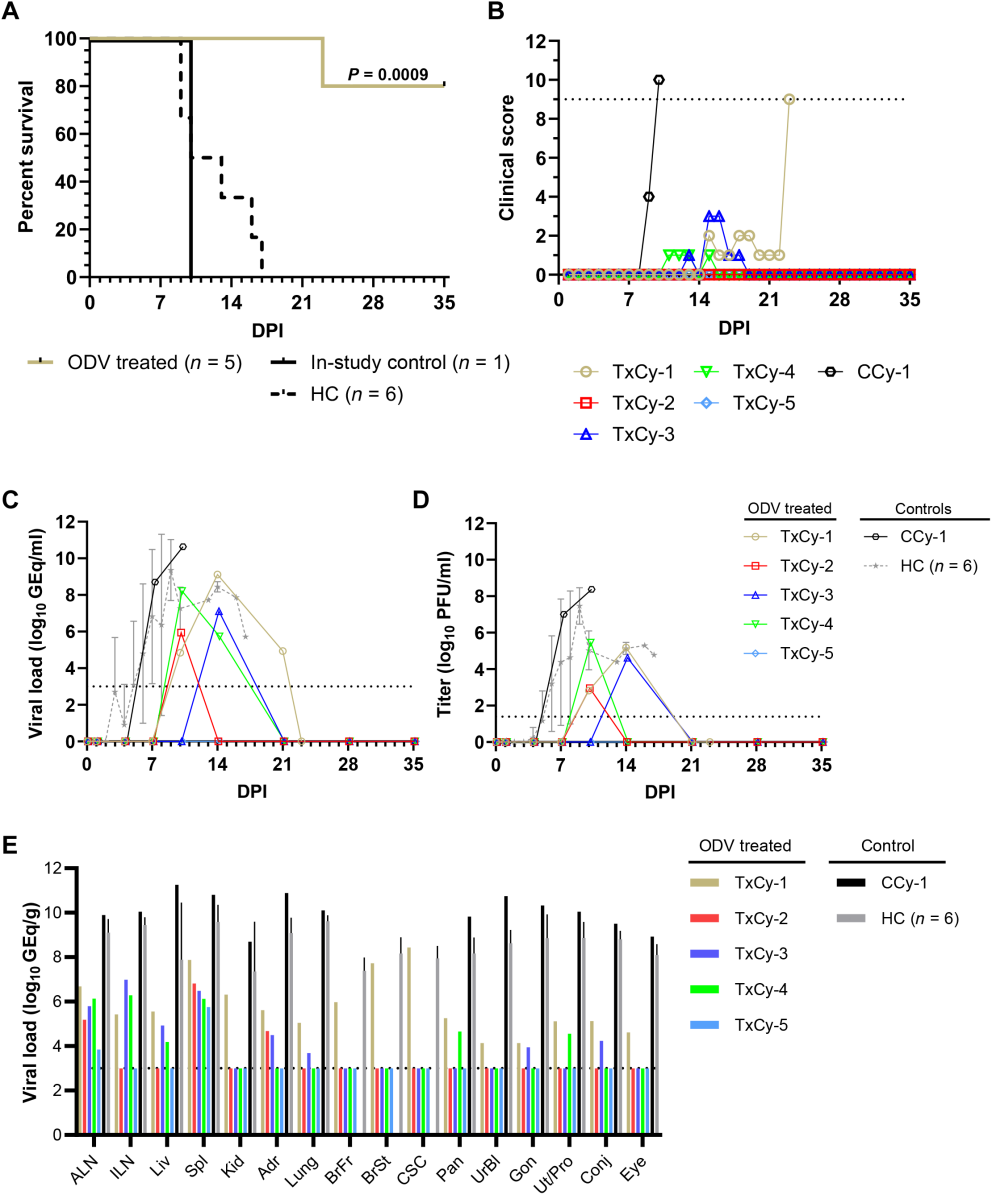
Survival analysis, clinical scoring, and virus replication kinetics in cynomolgus macaques challenged with EBOV and treated with ODV. (**A**) Kaplan-Meier survival curves for EBOV-challenged (Makona variant) cynomolgus macaques. The in-study control is plotted separately; however, for statistical comparison, the in-study control was grouped with the historical controls (HC) from a previous study (*14*). Differences in curves were tested by the Mantel-Cox log-rank test. (**B**) Clinical scores were assigned on the basis of daily cage-side observations of behavior and apparent physical health. The horizontal dashed line indicates the minimum clinical score by which euthanasia criteria were met. (**C** and **D**) Viral load was determined by RT-qPCR of RNA from whole blood (C) or plaque titration of plasma (D) collected at predetermined sampling points or at euthanasia. For both panels, individual data points represent the mean of two technical replicates. For HC, the geometric mean ± geometric SD for the cohort is plotted. To fit on a log scale axis, zero values [below limit of quantitation (LOQ)] are plotted as “1” (10^0^). (**E**) Viral load in selected tissues harvested at necropsy as determined by RT-qPCR detection of vRNA. The geometric mean titer ± SD is plotted for HC animals. For (C) to (E), dashed horizontal lines indicate the lower limit of quantitation (LLOQ) for the assay (1000 GEq/ml for RT-qPCR; 25 PFU/ml for plaque titration). To fit on a log scale axis, zero values (below LLOQ) are plotted.

To assess the postexposure protective efficacy of ODV, we first challenged a group of cynomolgus monkeys (*n* = 6) with a target dose of 10,000 PFU of EBOV (Makona variant) via the conjunctiva. Beginning 1 day postinfection (DPI), the experimental group (*n* = 5) received once-daily oral ODV suspension (100 mg/kg) for a total of 10 consecutive days of treatment. One animal served as an in-study placebo positive control and was treated in parallel with vehicle. The in-study placebo control (CCy-1) developed overt clinical signs of disease at 8 DPI which progressed to severe EVD, and the animal succumbed 10 DPI (Fig. 1, A and B, and table S1). This positive control animal showed clinical signs consistent with historical controls (table S1) (*14*) and exhibited marked deviations in hematological and serum chemistry parameters compared to baseline (day of challenge) values. These parameters included lymphocytopenia, thrombocytopenia, hypoalbuminemia, and elevated markers of hepatic/kidney injury [e.g., alanine aminotransferase (ALT), aspartate aminotransferase (AST), gamma-glutamyltransferase (GGT), blood urea nitrogen (BUN), and creatinine (CRE)] and acute systemic inflammation [C-reactive protein (CRP)] (table S1). Three of the five ODV-treated animals became febrile and developed petechial rashes, while one ODV-treated cynomolgus monkey showed no overt signs of disease, and another animal had only mild clinical illness (table S1). All five ODV-treated animals had changes in clinical pathology markers consistent with EVD including lymphopenia and thrombocytopenia (table S1). Four of the five ODV-treated animals survived the EBOV challenge and were healthy at the study endpoint (35 DPI), while one of the ODV-treated macaques (TxCy-1) had a protracted disease course that progressed to severe neurological complications resulting in euthanasia of this animal at 23 DPI (table S1). Of note, the ODV-treated animal that succumbed to disease at 23 DPI had no detectable virus in plasma, but viral RNA (vRNA) was detected in several tissues, including the forebrain, brainstem, and cervical spinal cord. For statistical comparison, the in-study positive control animal was grouped with the six aforementioned historical controls challenged with the same EBOV stock and dose via the conjunctival route (*14*) [*n* = 7, mean time to death (MTD) = 12.0 ± 3.1 DPI]. There was a significant difference in both the survival curves (*P* = 0.001; Mantel-Cox log-rank test) and the proportional survival (*P* = 0.01; Fisher’s exact test) between the ODV-treated and control groups (Fig. 1A). ODV treatment also delayed viral replication and reduced plasma viremia; at 7 DPI, ODV-treated animals had no detectable vRNA or infectious virus in plasma, when viral titers ranged from 5.35 to 7.0 log_10_ PFU/ml in control animals (Fig. 1, C and D).

### Virulence of EBOV in rhesus monkeys exposed by the conjunctival route

**Fig. 2.**
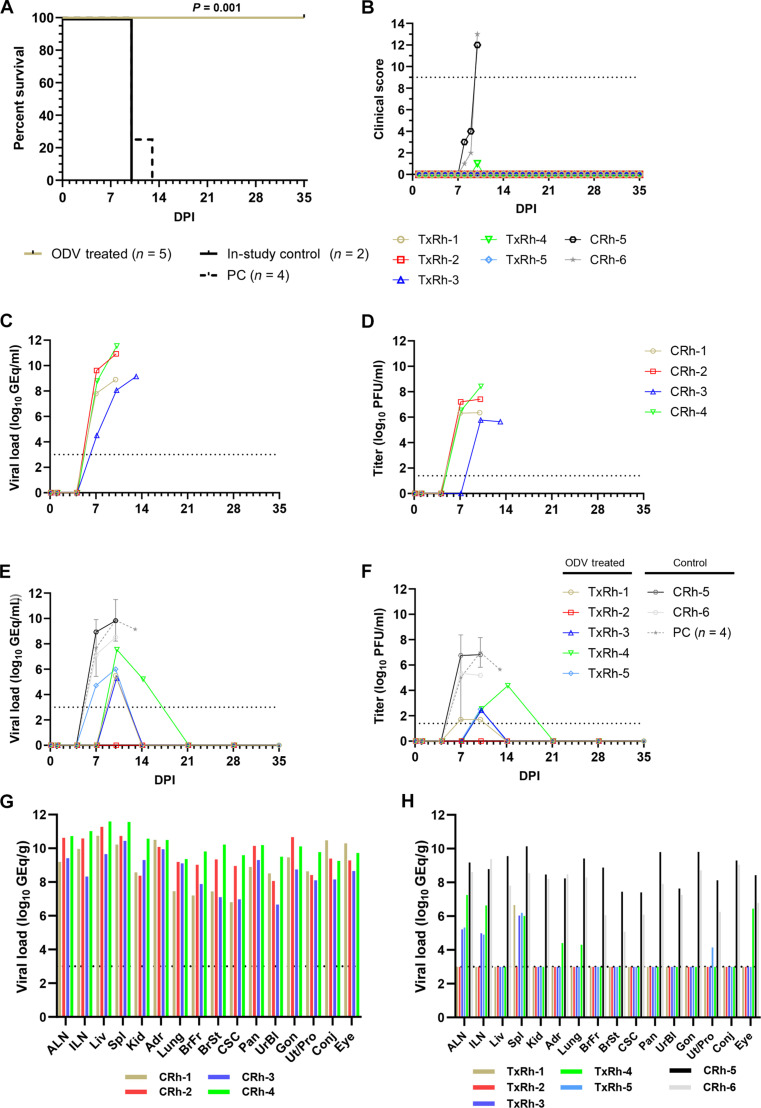
Survival analysis, clinical scoring, and virus replication kinetics in rhesus macaques challenged with EBOV and treated with ODV. (**A**) Kaplan-Meier survival curves for EBOV-challenged (Makona variant) rhesus macaques. The in-study control for the ODV treatment study is plotted separately; however, for statistical comparison, the in-study control was grouped with the untreated animals from the pilot study (PC). Differences in curves were tested by the Mantel-Cox log-rank test. (**B**) Clinical scores were assigned on the basis of daily cage-side observations of behavior and apparent physical health. The horizontal dashed line indicates the minimum clinical score by which euthanasia criteria were met. (**C** to **F**) Viral load was determined by RT-qPCR of RNA from whole blood [(C) and (E)] or plaque titration of plasma [(D) and (F)] collected at predetermined sampling points or at euthanasia. For (C) to (F), individual data points represent the mean of two technical replicates. Data plotted for PC represents the geometric mean ± geometric SD for the cohort. To fit on a log scale axis, zero values (below LOQ) are plotted as 1 (10^0^). (**G** and **H**) Viral load in selected tissues harvested at necropsy from untreated animals in the pilot study (G) and ODV-treated animals in the second study (H) as determined by RT-qPCR detection of vRNA. For (C) to (H), horizontal dashed lines indicate the LLOQ for the assay (1000 GEq/ml for RT-qPCR; 25 PFU/ml for plaque titration). To fit on a log scale axis, zero values (below LLOQ) are plotted as 1 (10^0^).

As we observed promising but incomplete protection in ODV-treated cynomolgus monkeys infected by the conjunctival route, we hypothesized that protection may be better in rhesus monkeys as the disease course for EBOV in intramuscularly infected rhesus monkeys is slower than cynomolgus monkeys under near identical test conditions (*13*). However, as we have never tested the virulence of the Makona variant of EBOV by the conjunctival route in rhesus macaques, we first performed a pilot study in four monkeys to determine the pathogenic potential of EBOV (Makona variant) when administered by this means using the same 10,000 PFU dose that produces uniform lethality in cynomolgus monkeys (*14*). All four rhesus monkeys developed severe EVD with clinical signs and changes in clinical pathology markers consistent with disease observed in cynomolgus monkeys (*14*), with animals succumbing on 10, 10, 10, and 13 DPI, respectively (MTD = 10.75 ± 1.3 DPI) (Fig. 2, A and B, and table S2). To determine whether there was a difference in the disease course between NHP species, we pooled the survival data of the four rhesus macaques in this pilot study with two positive control animals from a subsequent ODV treatment study (CRh-5 and CRh-6, described below) and compared the survival curves to those of the control cynomolgus macaque from the earlier ODV treatment study pooled with the six historical controls from a previously published study (*14*). There was no significant difference in the survival curves between the two species (*P* = 0.521, Mantel-Cox log-rank test) (fig. S1A). However, circulating EBOV vRNA was detected significantly earlier in cynomolgus macaques compared to rhesus macaques (*P* = 0.0210, Mann-Whitney *U* test; fig. S1B), suggesting that the kinetics of infection differ between these species under near identical test conditions. A limitation of this analysis is that blood from the cynomolgus macaque historical controls was sampled daily from 0 to 8 DPI (*14*), whereas the rhesus macaques in the current study were not. While the earliest detection of circulating EBOV vRNA in rhesus macaques was uniformly 7 DPI, we did not sample blood from these animals at 5 or 6 DPI; therefore, it is possible that detectable vRNA may have been present in some or all animals at these time points, which would affect the statistical significance of this analysis. However, it should be noted that EBOV vRNA was detected in three of seven (43%) cynomolgus macaques at 3 DPI (*14*), while all rhesus macaques were free of detectable vRNA at 4 DPI in the present study (fig. S1B), indicating that some replicative advantage for the virus in the former species may exist following challenge via the conjunctival route. These data comport with observed differences in the progression of disease between these species following intramuscular inoculation (*13*).

### Experimental challenge of rhesus macaques with EBOV and treatment with ODV

To assess the postexposure protective efficacy of ODV in EBOV-infected rhesus monkeys, we challenged a group of macaques (*n* = 7) with a target dose of 10,000 PFU of EBOV (Makona variant) via the conjunctiva. Beginning 1 DPI, the experimental group (*n* = 5) received once-daily oral doses (100 mg/kg) of ODV for a total of 10 consecutive days of treatment. One animal served as an in-study placebo positive control and was treated in parallel with vehicle, while a second in-study positive control animal was untreated. The placebo control (CRh-5) and the untreated positive control (CRh-6) developed overt clinical signs of disease at 6 DPI and 7 DPI, respectively, which progressed to severe EVD, and both control animals succumbed on 10 DPI (*n* = 2, MTD = 10.0 ± 0.0 DPI) (Fig. 2, A and B, and table S3). The EBOV positive control animals showed clinical signs consistent with the four rhesus macaques in the pilot study above (tables S2 and S3) and exhibited similar deviations in hematological and serum chemistry parameters compared to baseline (day of challenge) values, i.e., lymphocytopenia, thrombocytopenia, and elevated markers of hepatic injury (e.g., ALT and AST) and acute systemic inflammation (CRP) (table S3). While four of five ODV-treated rhesus monkeys developed transient fever, clinical signs of disease in all five animals were mild (table S3). In contrast to the petechial rashes observed in our initial ODV study in cynomolgus macaques, none of the ODV-treated rhesus monkeys exhibited petechial rashes, and all five survived and were healthy at the predetermined 35 DPI study endpoint (Fig. 2A and table S3). All five ODV-treated rhesus macaques had some transient perturbations in clinical pathology markers consistent with but less pronounced than those we observed in the ODV-treated cynomolgus monkeys (table S3). As in the previous study, for statistical comparison, the two in-study controls from this ODV treatment study were grouped with the four animals from the rhesus pilot study (*n* = 6, MTD = 10.5 ± 1.1 DPI). There was a significant difference in the survival curves (*P* = 0.001; Mantel-Cox log-rank test) between the ODV-treated and control groups (Fig. 2A).

### Reduction of viral load

As treatment was initiated 1 DPI in both macaque studies, all animals, including the in-study positive controls, had undetectable levels of vRNA or infectious EBOV at the time ODV was first administered, as measured by real-time quantitative polymerase chain reaction (RT-qPCR) of whole blood or plaque titration of plasma, respectively (Figs. 1, C and D, and 2, C to F). In the first treatment study in cynomolgus macaques, one animal (TxCy-5) remained free of detectable quantities of circulating vRNA or infectious EBOV through the study endpoint (Fig. 1, C and D). Low to moderate levels of vRNA [5.73 to 8.21 log_10_ genome equivalents (GEq)/ml] were transiently detected in subject TxCy-2 at 10 DPI, TxCy-3 at 14 DPI, and TxCy-4 at 10 and 14 DPI. Likewise, low to moderate titers (2.38 to 5.48 log_10_ PFU/ml) of circulating infectious virus were transiently detected in subjects TxCy-2 and TxCy-4 at 10 DPI and TxCy-3 at 14 DPI. In contrast, the vehicle-treated control cynomolgus monkey CCy-1 became viremic at 7 DPI with moderate to high quantities of vRNA (8.71 to 10.64 log_10_ GEq/ml) and high levels of circulating infectious virus (7.00 to 8.38 log_10_ PFU/ml) detected through to the terminal phase of disease (Fig. 1, C and D). The single ODV-treated cynomolgus monkey in the first treatment study that succumbed on 23 DPI (TxCy-1) had low level viremia on 10 DPI (4.85 GEq/ml; 2.83 log_10_ PFU/ml) that peaked on 14 DPI (9.21 GEq/ml; 5.18 log_10_ PFU/ml), with only low levels of vRNA on 21 DPI and no vRNA or circulating infectious EBOV detected on 23 DPI (Fig. 1, C and D). For statistical comparison, vRNA abundance and plaque titers from the in-study control animal were pooled with data from the six historical control animals from a previous study. The peak viral burden, whether measured by RT-qPCR or plaque titration, was significantly lower in ODV-treated animals compared to the control cohort (*P* = 0.018 for both comparisons, Mann-Whitney *U* test) (fig. S2, A and B).

In the study to assess the pathogenic potential of EBOV in rhesus monkeys by the conjunctival route, a range of low to high levels of vRNA (4.51 to 9.61 log_10_ GEq/ml) was first detected in whole blood of all four animals on 7 DPI, with titers peaking as high as 11.54 GEq/ml by the terminal time point for each animal (Fig. 2C). Likewise, high titers (6.31 to 7.20 log_10_ PFU/ml) of circulating infectious virus were detected in three of four rhesus monkeys at 7 DPI and all four macaques by the terminal time point for each animal (Fig. 2D).

In the second treatment study in rhesus macaques, one animal (TxRh-2) remained free of detectable quantities of circulating vRNA or infectious EBOV through the study endpoint (Fig. 2, E and F). Low to moderate levels of vRNA (4.72 to 7.54 log_10_ GEq/ml) were transiently detected in subjects TxRh-1, TxRh-3, and TxRh-5 at 10 DPI and TxRh-4 at 7, 10, and 14 DPI. Likewise, low to moderate titers (1.70 to 4.37 log_10_ PFU/ml) of circulating infectious virus were transiently detected in subjects TxRh-1 and TxRh-4 at 7 and 10 DPI and TxRh-3 and TxRh-4 at 10 DPI. In contrast, the two in-study positive control rhesus monkeys became viremic at 7 DPI with moderate to high quantities of vRNA (8.52 to 9.82 log_10_ GEq/ml) and moderate to high levels of circulating infectious virus (5.35 to 6.81 log_10_ PFU/ml) detected through to the terminal phase of disease (Fig. 2, E and F). For statistical comparison, vRNA abundance and plaque titers from the two in-study control animals were pooled with data from the four untreated animals from the pilot study in rhesus macaques. The peak viral burden, whether measured by RT-qPCR or plaque titration, was significantly lower in ODV-treated animals compared to the control cohort (*P* = 0.004 for both comparisons, Mann-Whitney *U* test) (fig. S2, C and D).

Tissues were harvested at necropsy from all cynomolgus monkeys in the first treatment study and assayed for vRNA by RT-qPCR. Low to moderate levels of vRNA (3.94 to 6.99 log_10_ GEq/g tissue) were detected mostly in lymphoid tissues of all four surviving ODV-treated animals (Fig. 1E), in contrast to the high levels (~ 9 to 11 log_10_ GEq/g tissue) which were found in all tissues screened from the vehicle-treated control cynomolgus monkey. Low to moderate levels of vRNA (4.14 to 8.43 log_10_ GEq/g tissue) were detected in all tissues of the ODV-treated cynomolgus monkey that succumbed on 23 DPI with the highest level detected in the spleen, brain stem, and cervical spinal cord. We were unable to detect any infectious EBOV by plaque titration in the spleen or brain stem of this animal. In the study to assess the pathogenic potential of EBOV in rhesus monkeys by the conjunctival route, moderate to high levels of vRNA (~ 7 to 12 log_10_ GEq/g tissue) were found in all tissues screened (Fig. 2G). In the second treatment study in rhesus monkeys, low to moderate levels of vRNA (3.94 to 6.99 log_10_ GEq/g tissue) were detected mostly in lymphoid tissues of four of five surviving ODV-treated animals, while no vRNA was detected in any tissues of ODV-treated animal TxRh-2 (Fig. 2H). In contrast, high levels (~ 9 to 10 log_10_ GEq/g tissue) were found in target tissues (liver and lymphoid tissues) of the two in-study positive control rhesus monkeys (Fig. 2H).

### Assessment of neutralizing antibody activity by PRNT_50_

For all studies, the presence of anti-EBOV neutralizing antibodies was assessed by plaque reduction neutralization test (PRNT) using serum collected prechallenge (0 DPI), 14 DPI, and before euthanasia due to terminal disease or at the study endpoint (35 DPI) (fig. S3, A to C). As expected, prechallenge serum uniformly failed to achieve 50% reduction in EBOV titers compared to the virus control plate. For the ODV treatment study in cynomolgus monkeys, neutralization capacity was weak to nonexistent, with a maximum endpoint neutralizing antibody titer (expressed as the reciprocal of the last dilution at which at least 50% neutralization was observed) of 20 being reached in a single animal (TxCy-2) by the study endpoint (35 DPI) (fig. S3A). Only two of four rhesus macaques from the pilot study exhibited any detectable virus neutralization at the terminal time point (10 or 13 DPI), and only subject CRh-3 reached 50% neutralization at a titer of 10 (fig. S3B). Similar results were observed in the ODV treatment study in rhesus monkeys, with three of five treated animals achieving endpoint titers of 20 by the end of the study (35 DPI) (fig. S3C).

### Assessment of binding antibody titers by ELISA

**Fig. 3.**
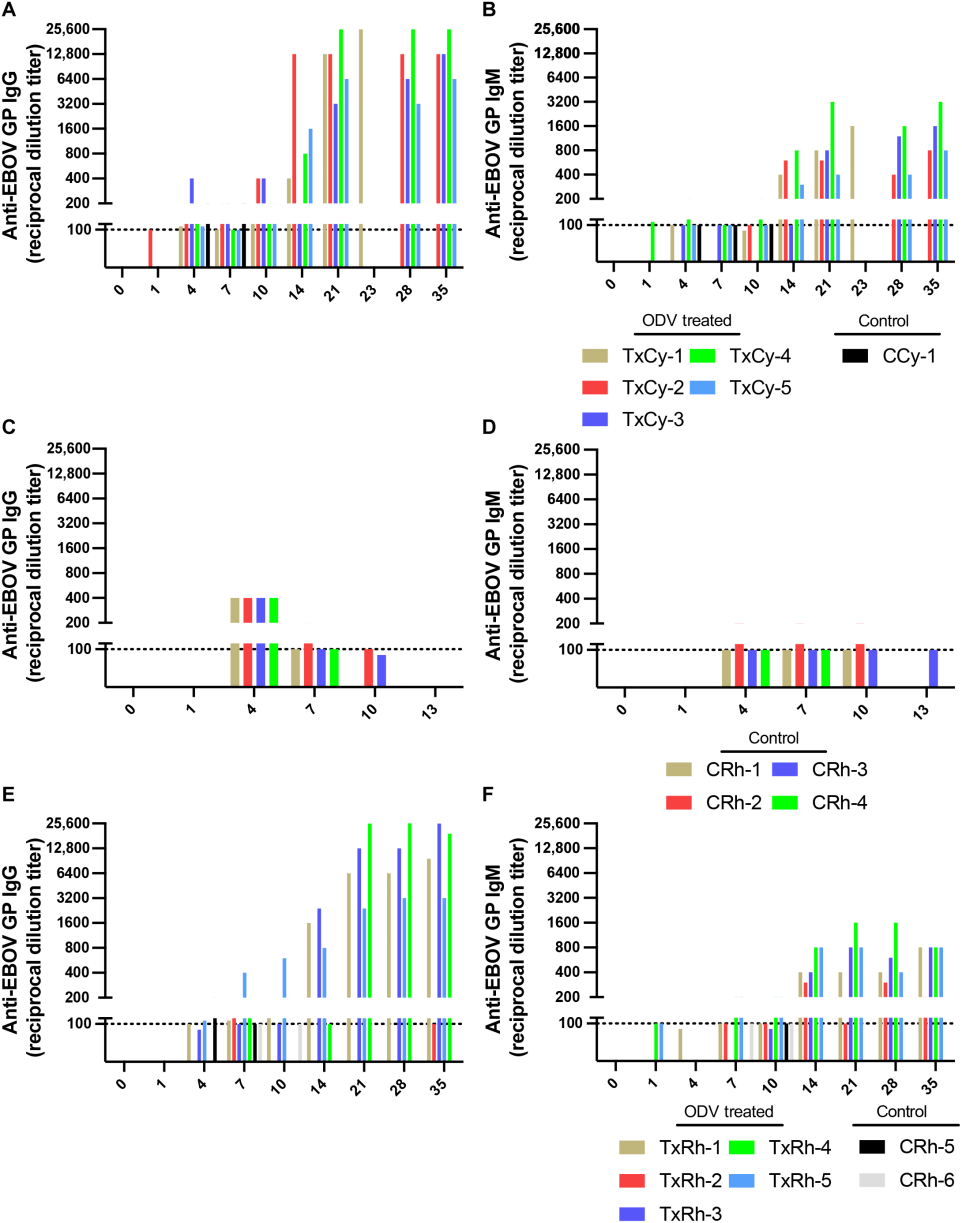
Anti-EBOV GP antibody titers in control and ODV-treated macaques. Plasma samples from each study [(**A** and **B**) ODV cynomolgus macaque (*N* = 6); (**C** and **D**) pilot rhesus (*N* = 4); (**E** and **F**) ODV rhesus macaque (*N* = 7)] were tested for circulating anti-EBOV GP-specific IgG [(A), (C), and (E)] and IgM [(B), (D), and (F)] binding antibody responses by indirect ELISA. Line graphs (log_2_ *y* axis) depicting the average reciprocal dilution titer for individual subjects at each time point (0, 1, 4, 7, 10, 14, 21, 28, terminal, or 35 DPI) are shown. To fit on a log scale axis, zero values are plotted as 1 (10^0^). The dotted line (1:100 dilution) represents the limit of detection for the assay.

According to our enzyme-linked immunosorbent assay (ELISA) results, ODV-treated subjects developed substantial anti-EBOV glycoprotein-specific immunoglobulin M (IgM) and IgG binding antibodies, exceeding titers of 1:100 by 14 DPI (Fig. 3, A to F). For both NHP species, IgM titers in treated subjects were detectable at 14 DPI and remained low (1:300 to 1:3200) throughout the course of the study (Fig. 3, B and F). For the cynomolgus macaque study, five of five ODV-treated subjects developed antibodies that were not consistently observed until after 10 DPI, with peak IgG titers ranging from 1:6400 to 1:25,600 (Fig. 3A). The single subject (TxCy-1) that developed severe neurological complications and was euthanized at 23 DPI also developed robust IgG binding titers (1:25,600) before death. For the rhesus macaque study, four of five ODV-treated subjects developed substantial IgG titers ranging from 1:3200 to 1:25,600 that were also not consistently observed until after 10 DPI (Fig. 3E). Nonexistent to weak IgG and IgM titers were observed in TxRh-2 (Fig. 3, E and F), which was consistent with the PRNT findings for this particular subject (fig. S3C). Overall, the magnitude and kinetics of the anti-glycoprotein (GP) IgM and IgG response in ODV-treated animals was similar to what has been previously reported in rhesus macaques vaccinated with the recombinant VSV-ZEBOV vaccine as a PEP (*15*), suggesting that ODV-mediated viral suppression is not affecting virus-specific humoral immune response.

### Gross lesions and histopathology

In the initial study to assess the protective efficacy of ODV in cynomolgus monkeys, gross and histologic lesions consistent with the EVD conjunctival model (*14*) were observed exclusively in the in-study positive control animal (CCy-1). Lymphoid lesions included sinus histiocytosis, fibrin deposition, and germinal center necrosis. Anti-EBOV antigen was detected in mononuclear cells throughout the lymphoid tissues (fig. S4B). In representative gastrointestinal tissue sections, lesions ranged from lymphohistiocytic inflammatory infiltrates to multifocal hepatic necrosis. Immunohistochemistry (IHC) positivity was noted in Kupffer cells, hepatic sinusoidal lining cells, and occasionally hepatocytes (fig. S4A). In the intestinal tract, EBOV antigen was detected in mononuclear cells within the lamina propria, rarely overlying epithelial cells, and scattered interstitial mononuclear cells in the pancreas and salivary gland.

Lymphohistiocytic interstitial inflammatory infiltrates were also observed within urogenital tissues, including the kidney, urinary bladder, epididymis, testis, and prostate. Positive IHC was noted in the endothelium and mononuclear cells within the interstitium of these organs. In pulmonary tissues, minimal alveolar flooding with edema was evident, with an increased number of alveolar macrophages and thickened alveolar septae with lymphohistiocytic infiltrates. IHC positivity for anti-EBOV antigen was observed in the alveolar septum, alveolar macrophages, and occasionally the endothelium. Adrenalitis was noted on hematoxylin and eosin (H&E) with associated IHC positivity in scattered mononuclear cells and clusters of cortical cells. The ODV-treated animal with a protracted time to death (TxCy-1) and the four ODV-treated survivors exhibited no EVD-specific lesions or IHC positivity in lymphoid organs (fig. S4, E and H), gastrointestinal tissues (fig. S4, D and G), or pulmonary tissues. The ODV-treated macaque with a protracted disease course (TxCy-1) developed chronic renal inflammatory lesions but lacked associated IHC positivity for anti-EBOV antigen. No urogenital lesions or IHC positivity was noted in the four ODV-treated surviving animals.

Ocular lesions were present in both the in-study positive control (CCy-1) and the ODV-treated animal with a protracted disease course (TxCy-1). The in-study positive control (CCy-1) exhibited ocular lesions consistent with acute EVD, characterized by a modest lymphohistiocytic inflammatory infiltrate in the dermis of the conjunctiva/palpebrae and the uveal tract (primarily the choroid and ciliary body). IHC positivity was observed in the endothelium and mononuclear cells within the dermis of the conjunctiva/palpebrae and the uveal tract (fig. S4C). The ODV-treated animal TxCy-1 displayed moderate widespread lymphohistiocytic inflammatory infiltrates in the iris leaflets, anterior and posterior chambers, drainage angle, anterior portion of the vitreous chamber, and retina. Scattered mononuclear cells within the anterior portion of the vitreous chamber were IHC positive (fig. S4I). No lesions or IHC positivity was detected in the four ODV-treated surviving macaques (fig. S4F). In addition to widespread ocular inflammation, TxCy-1 had multifocal lesions in the nervous tissues. Representative sections from the frontal lobe, brainstem, temporal lobe, parietal lobe, and cervical spinal cord revealed one or more of the following lesions: glial nodules, lymphocytic perivascular cuffs, and/or multifocal degenerative/necrotic encephalitis (fig. S4, J and M). Positive IHC immunolabeling colocalized with lesions in the brainstem, temporal lobe, and cervical spinal cord (fig. S4, K, N, and O), and positive in situ hybridization (ISH) was also noted in representative neurolesions (fig. S4L). No lesions or IHC positivity was detected in the brain of the four surviving NHPs. Brain tissue was not collected for the in-study positive control animal.

In the pilot study to assess the pathogenic potential of EBOV in rhesus monkeys and the ODV treatment study in rhesus monkeys, lesions and immunolabeling in representative tissues were consistent with EVD in all six positive control animals (CRh1 to CRh6). The five ODV-treated survivors exhibited no lesions or immunolabeling consistent with EVD. Lymphoid lesions in the six positive control animals included sinus histiocytosis, fibrin deposition, and germinal center necrosis. Positive IHC for anti-EBOV antigen was noted in mononuclear cells throughout the lymphoid tissues (fig. S5B). In representative gastrointestinal tissue sections, lesions ranged from lymphohistiocytic inflammatory infiltrates to multifocal erosions and/or necrosis. Anti-EBOV antigen was noted in Kupffer cells, hepatic sinusoidal lining cells, and rarely in hepatocytes (fig. S5A, inset). In the intestinal tract, IHC positivity was also noted in the endothelium, mononuclear cells within the lamina propria, rarely overlying epithelial cells, and scattered interstitial mononuclear cells within the pancreas and salivary gland.

Urogenital lesions in the six EBOV-positive control rhesus monkeys included lymphohistiocytic interstitial inflammatory infiltrates of the kidney, urinary bladder, epididymis, testis, prostate, ovary, and uterus. Antigen was noted in the endothelium and mononuclear cells within the interstitium of these organs (fig. S5E). In pulmonary tissues, alveolar septae were thickened with lymphohistiocytic infiltrates. Minimal alveolar flooding with edema occurred, and there was an abundance of alveolar macrophages. IHC positivity was observed in the alveolar septum, alveolar macrophages, and occasionally the endothelium. Adrenalitis was noted on H&E with antigen within scattered mononuclear cells and clusters of cortical cells. Ocular lesions consistent with acute EVD were characterized by a modest lymphohistiocytic inflammatory infiltrate in the dermis of the conjunctiva/palpebrae and the uveal tract (primarily the choroid and ciliary body). IHC positivity was detected in the endothelium, mononuclear cells, and rarely in sebocytes within the dermis of the conjunctiva/palpebrae and the uveal tract (fig. S5, C and D). Neurological lesions in acute EVD included mild lymphohistiocytic infiltrates of the trigeminal ganglion and/or partial fibrin thrombi with hemorrhage in the choroid plexus. IHC positivity was observed in the endothelium, intravascular mononuclear cells, and rarely in ependymal cells and satellite cells of the trigeminal ganglion (fig. S5F, inset). No lesions or IHC positivity was noted in the five ODV-treated rhesus macaques [fig. S5, G (inset) and H to L (inset)]. In summary, lesions and IHC positivity in tissues of positive control cynomolgus and rhesus monkeys were comparable as were the lack of lesions and IHC positivity in ODV-treated animals with the exception of the ODV-treated cynomolgus monkey with the protracted disease course as described above.

### Transcriptomics

To identify potential immune correlates associated with ODV-mediated protection, we conducted targeted transcriptomics on whole blood RNA samples from EBOV-exposed cynomolgus (*N* = 6; 5 treated, 1 in-study control) and rhesus macaques (*N* = 11; 5 treated, 2 in-study controls, and 4 pilot study controls) (data S1). Principal components analysis was applied at each selected DPI (0, 4, 7, and 10 DPI) for dimensionality reduction to evaluate the contribution of four parameters—disease severity (none, mild, and severe), final disposition (fatal and survivor), treatment (ODV and control), and NHP species—to the overall variability within the dataset (fig. S6). No distinct separation was observed for the disease severity parameter. Minimal spatial variation was noted for the disposition and treatment parameters until 10 DPI, when these parameters began to drive most transcriptional changes. At 0, 4, and 7 DPI, transcriptional variation was primarily attributed to NHP species; therefore, we analyzed each NHP species separately for further host response investigation.

**Fig. 4.**
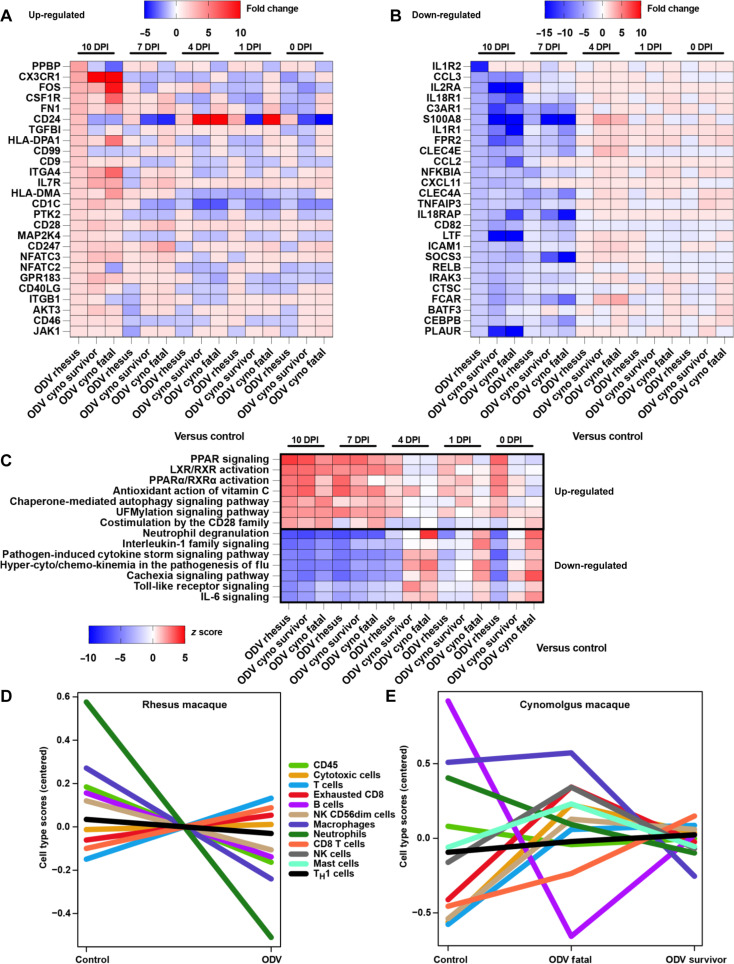
Transcriptional profiling of ODV-treated versus control macaques exposed to EBOV. RNA samples from all subjects [*N* = 6 cynomolgus macaques (*N* = 5 treated including 1 fatal subject and *N* = 1 in-study control subject); *N* = 11 rhesus macaques (*N* = 5 treated all survivors and *N* = 6 control subjects with two in-study controls and four pilot study controls] were included at the following time points: 0, 4, 7, and 10 DPI. Groups: Rhesus control (*N* = 6), rhesus ODV (*N* = 5), cyno control (*N* = 1), cyno fatal (*N* = 1), cyno survivor (*N* = 4). Heatmaps of the most up-regulated (**A**) and down-regulated (**B**) transcripts in whole blood from ODV-treated versus control macaques exposed to EBOV. Any differentially expressed genes (DEGs) with a Benjamini-Hochberg FDR corrected *P* value less than 0.05 were deemed significant; the dataset was filtered by the ODV rhesus group at 10 DPI in order of fold change. Red indicates increased expression; blue indicates decreased expression; white indicates no change in expression. (**C**) Ingenuity Pathway Analysis–based enrichment of transcripts in ODV-treated versus control macaques exposed to EBOV at 0, 4, 7, and 10 DPI. Up-regulated pathways were classified by a >1.5 *z* score, and down-regulated pathways were classified by a <−1.5 *z* score. Pathways are sorted by the topmost positive (top) or negative (bottom) *z* score for the ODV rhesus group at 10 DPI. (**D** and **E**) Immune cell transcriptional profiling of controls and ODV-treated rhesus (D) and cynomolgus (E) macaques exposed to EBOV. Higher cell type scores indicate a higher abundance of transcripts mapping to the specific cell subset. All samples (0, 4, 7, and 10 DPI) were combined to generate cell type quantities for each group. Cyno, cynomolgus macaque group; T_H_1, T helper cell 1.

Given that dimensional separation for the treatment variable (ODV and control) emerged at 10 DPI, we focused subsequent analyses on this time point. Differential expression analysis [false discovery rate (FDR)–corrected *P* < 0.05] revealed that ODV-treated macaques showed higher expression of transcripts involved in antigen presentation (*HLA-DP1*, *HLA-DMA*, *CD40LG*, *GPR183*, and *CD247*) and lymphocyte development and activation [*CD99*, *CD9*, *CD28*, *IL7R*, nuclear factor of activated T cells, cytoplasmic 3 (*NFATC3*), and *NFATC2*] (Fig. 4A). Notably, *CX3CR1* was the most highly expressed transcript across ODV-treated groups, which guides immune cells such as monocytes, natural killer cells (NK cells), and T cell subsets to sites of inflammation (*16*). CD99 and IL7R are critical for T cell maturation in the thymus (*17*, *18*), while CD9 contributes to immune synapse formation and platelet aggregation, essential for blood clotting (*19*, *20*). CD28 enhances T cell activation by amplifying signals through its ligands CD80/CD86 (*21*). NFATC3 and CD247 are integral to T cell activation and cytokine production (*22*, *23*), with CD1c contributing to antigen presentation (*24*, *25*).

In contrast, down-regulated transcripts in fatal cases included those linked to interleukin-1 (IL-1) signaling (*IL1R2*, *IL18R1*, *IL1R1*, and *IL18RAP*) (*26*–*28*), monocyte/macrophage chemokine signaling (*CCL3* and *CCL2*) (*29*), and C-type lectin cell adhesion (*CLEC4E* and *CLEC4A*) (Fig. 4B) (*30*, *31*). Many of these molecules were initially up-regulated until about 7 DPI but declined later, when ODV-treated subjects seem to diverge from control subject transcriptional signatures.

Differences between the ODV-treated fatal subject (TxCy-1) and survivors highlighted *NFATC2* and *ITGB1* as potentially critical (Fig. 4A). *ITGB1*, encoding integrin β1, is vital for cell adhesion, migration, signaling, and the differentiation of lymphocytes (*32*). NFATC2 plays a pivotal role in the regulation of T cell activation and differentiation (*33*–*35*). It functions as a transcription factor that translocates to the nucleus upon T cell receptor activation, where it regulates the expression of genes critical for immune responses, including cytokines such as IL-2, which is essential for T cell proliferation and survival. NFATC2 is involved in controlling the balance between effector T cells, which are responsible for attacking pathogens, and regulatory T cells, which help prevent autoimmunity. In the context of successful ODV treatment, increased expression of ITGB1 and NFATC2 suggests enhanced T cell activity, contributing to a robust and regulated immune response.

Functional enrichment analysis of transcripts identified peroxisome proliferator–activated receptor (PPAR) signaling as the most up-regulated pathway in treated versus control subjects (Fig. 4C). PPAR-specific ligands have been shown to inhibit the production of proinflammatory cytokines, such as IL-6, tumor necrosis factor–α (TNF-α), IL-1β, and nitric oxide in various cell types (*36*, *37*), a potential mechanism by which excessive inflammation is controlled/modulated by early ODV treatment. Other up-regulated pathways in the treated groups included chaperone-mediated autophagy signaling, UFMylation signaling [recently discovered posttranslational modification process that involves the conjugation of a small ubiquitin-like protein (*38*)], and T cell receptor CD28 costimulation signaling. Conversely, down-regulated pathways were those typically associated with EVD, including neutrophil degranulation, IL-1 signaling, cytokine storm signaling, and hypercytokinemia/hyperchemokinemia signaling.

To detect shifts in circulating immune cell populations, digital cell quantitation was performed via transcriptional profiling (Fig. 4, D and E). In line with the differential expression analysis, ODV treatment in rhesus macaques was associated with a predicted increase in T cells, CD8 T cells, and cytotoxic cells (Fig. 4D). In cynomolgus macaques, successful ODV treatment was linked to a predicted increase in these same cell subsets, along with NK cells (Fig. 4E). In contrast, the control groups showed a predicted abundance of neutrophils and macrophages. The single treated fatal subject (Tx-Cy1) had a predicted decrease in both B cells and CD8 T cells. These findings suggest that ODV-treated animals that survived EBOV challenge had enhanced markers of adaptive immune responses and decreased markers of excessive inflammation

### Effect of ODV treatment on plasma cytokines, chemokines, and coagulation markers

**Fig. 5.**
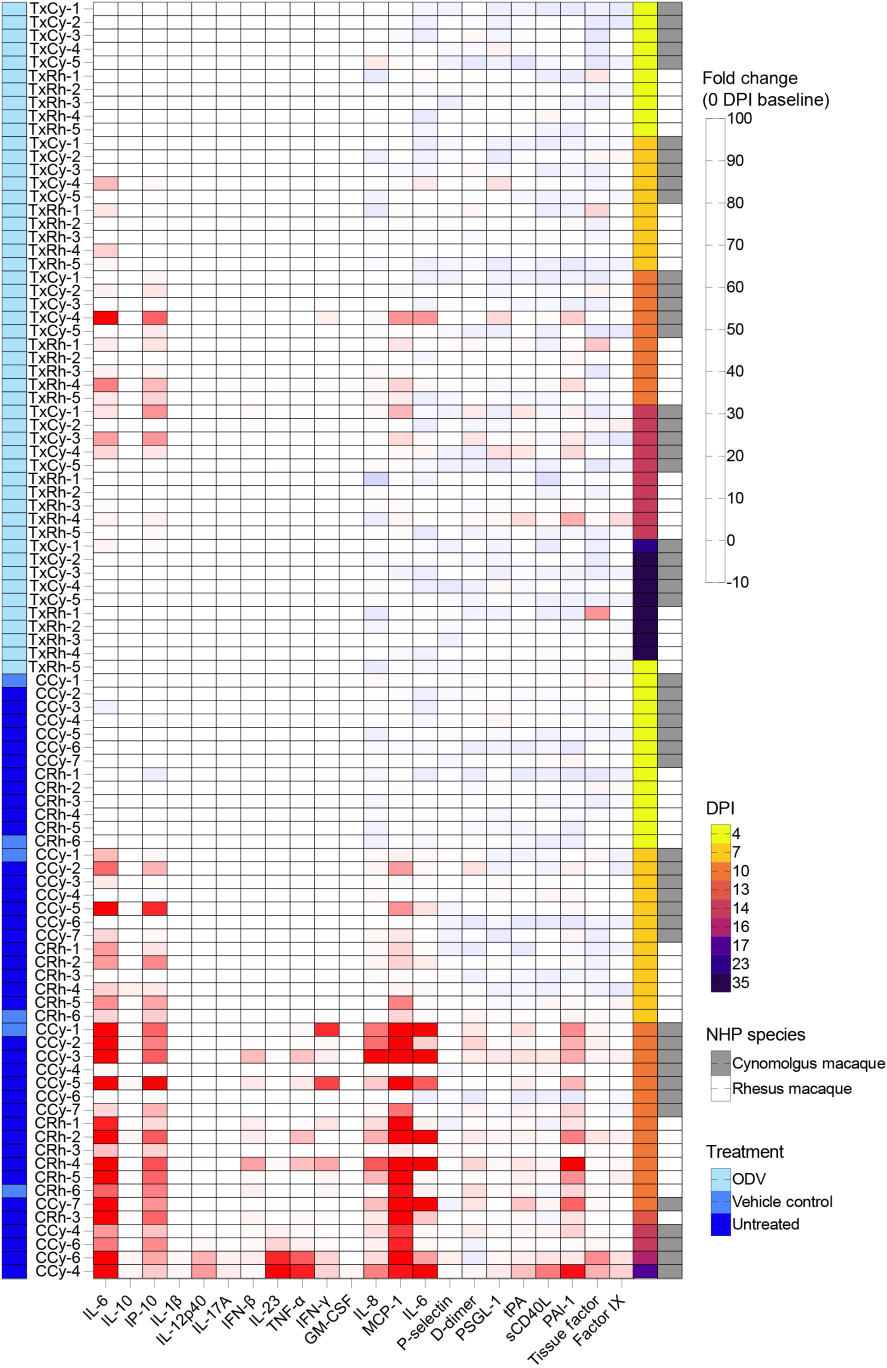
Plasma levels of cytokines/chemokines and coagulation markers in ODV-treated versus control macaques exposed to EBOV. Expression of circulating proinflammatory mediators and thrombosis analytes in the plasma of EBOV-exposed cynomolgus (*N* = 12) and rhesus (*N* = 11) macaques at 0, 4, 7, 10, and 35 DPI or the terminal time point. To help identify trends, the in-study cynomolgus macaque control subject (CCy-1) was grouped with historical controls (*N* = 6; CCy-2, CCy-3, CCy-4, CCy-5, CCy-6, and CCy-7) from a previous study that were exposed by the same route at the same target dose (*14*). Depicted are fold change values for each analyte with respect to a prechallenge baseline (0 DPI) for each subject. Red indicates increased expression; blue indicates decreased expression; white indicates no change in expression. Individual data points represent the mean of two technical replicates. GM-CSF, granulocyte-macrophage colony-stimulating factor.

To examine the host response following EBOV challenge and ODV treatment, we lastly measured protein levels of cytokines, chemokines, growth factors, and coagulation markers in the plasma of each subject. Samples from EBOV-exposed cynomolgus (*N* = 12) and rhesus (*N* = 11) macaques at 4, 7, 10, and 35 DPI or the terminal time point were compared against a prechallenge baseline (0 DPI) (Fig. 5). To help identify trends, the in-study cynomolgus macaque control subject (CCy-1) was grouped with historical controls (*N* = 6; CCy-2, CCy-3, CCy-4, CCy-5, CCy-6, and CCy-7) from a previous study in cynomolgus macaques that were exposed by the same route at the same target dose (*14*).

ODV treatment was associated with reduced expression of proinflammatory cytokines and coagulation markers in both NHP species. However, transient spikes in IL-6, interferon gamma-induced protein 10 (IP-10), monocyte chemoattractant protein 1 (MCP-1), and plasminogen activator inhibitor-1 (PAI-1) were observed in some subjects, which resolved by the 35 DPI study endpoint. In contrast, control groups exhibited a significant and sustained increase in IL-6, IP-10, TNF-α, IL-8, and MCP-1, accompanied by elevated levels of coagulation markers such as tissue-type plasminogen activator (tPA), D-dimers, and PAI-1, indicating an imbalanced immune response and a dysregulated clotting cascade (*39*). At 21 DPI, these markers were also elevated in the TxCy-1 subject, which was euthanized because of neurological complications at 23 DPI. These findings suggest that ODV treatment delayed, mitigated, or even prevented the onset of a cytokine storm and helped to avert coagulopathies.

## DISCUSSION

The devastating 2013–2016 epidemic of EBOV in West Africa and the recent severe acute respiratory syndrome coronavirus 2 (SARS-CoV-2) global pandemic emphasize the need for antiviral treatments to stop the spread of infectious diseases. For SARS-CoV-2, intravenous remdesivir was the first available intervention followed by mAb therapy. However, as both remdesivir and mAb treatment requires intravenous infusion, substantial resources were committed to developing oral antivirals such as molnupiravir and nirmatrelvir/ritonavir which have proven effective in combating COVID-19 (*40*). EBOV outbreaks, including the West African epidemic, have been characterized by high infection rates among the medical staff which severely compromised the capability of health clinics to treat patients. This problem is further complicated because the only approved treatments for diagnosed cases of EBOV are human mAbs, which necessitate special handling requirements as well as highly trained personnel to perform intravenous infusions, adding substantial burden to low resource settings. A medical countermeasure effective against all filoviruses that could be rapidly and potentially more easily administered (e.g., via the oral route) to immediately prevent or slow the infection and allow the patient more time to develop a protective response could change treatment paradigms during a filovirus outbreak. Also, vaccination can be an important component of an outbreak response particularly as was shown for ERVEBO (*41*). While vaccines have limitations (valency, storage requirements, administration by injection, and the delay from administration to mounting protective immune response, i.e., ~10 days), coadministration of ERVEBO and ODV during this period may bridge the interval during which patients are still susceptible to EVD.

Here, we show that ODV, an oral prodrug of nucleoside GS-441524, can protect 80% of cynomolgus monkeys and 100% of rhesus monkeys against lethal EBOV infection when administered 24 hours after virus exposure. While our data did not show a difference in MTD of cynomolgus versus rhesus monkeys under near identical test conditions, circulating viremia did appear to develop faster in cynomolgus monkeys than rhesus monkeys, which is consistent with previous studies suggesting a more rapid disease course in EBOV-infected cynomolgus versus rhesus monkeys (*12*, *13*). This quicker development of viremia in cynomolgus monkeys may in part explain the trend toward better protection of ODV in EBOV-infected rhesus monkeys. While most surviving animals had moderate amounts of EBOV vRNA in lymphoid and other tissues at the study endpoint, all tissues from surviving animals lacked IHC reactivity, and attempts to isolate infectious virus from several tissues from subject TxCy-1 with high vRNA loads were unsuccessful. Residual vRNA in the absence of infectious virus has been documented by us in filovirus (*10*, *42*) and arenavirus (*43*, *44*) vaccine and treatment studies as well as others for a number of different virus infection models (*45*) and likely relates to ongoing immune clearance mechanisms (i.e., neutralizing antibodies, cellular immunity, etc.).

In this study, we investigated potential immune correlates linked to ODV-mediated protection against EBOV in cynomolgus and rhesus macaques. Distinct transcriptional and protein changes emerged in ODV-treated animals. These animals exhibited higher expression of transcripts involved in antigen presentation and T cell activation, including *CX3CR1*, *CD99*, *IL7R*, *CD28*, *NFATC3*, and *CD247*, suggesting enhanced adaptive immunity. Conversely, fatal cases were associated with innate immune signaling and excessive monocyte/granulocyte-driven inflammation, which may have contributed to an inadequate immune response. Functional enrichment highlighted up-regulated pathways in ODV-treated animals, such as PPAR signaling, which is associated with reduced inflammation, while pathways linked to severe disease, including cytokine storm signaling, were down-regulated. Plasma analysis confirmed that animals treated with ODV generally had reduced proinflammatory cytokines and coagulation markers, although transient spikes were observed. These findings suggest that ODV treatment affords the opportunity for the development of adaptive immunity while mitigating excessive inflammation, potentially preventing fatal outcomes. While evidence of seroconversion was observed in all surviving animals with the exception of TxRh-2, antibody neutralization activity (as measured by PRNT) remained low to modest by the study endpoint. While antibody neutralization has been shown to be an important feature of vaccine-mediated immunity against EBOV infection in NHPs (*46*–*49*) and humans (*50*–*53*), classic kinetics of antibody responses are reported to not always occur during EBOV infection of humans (*54*–*57*), and other nonneutralizing roles for antibodies, such as Fc-mediated phagocytosis and NK cell activation, may play a role in protective immunity (*58*).

A major goal of this study was to determine whether the complete protection of NHPs that we recently reported for SUDV when ODV was administered beginning at 24 hours postexposure could be translated to other filoviruses, most importantly EBOV. Additional work is warranted to determine whether further treatment delay affects the efficacy of ODV as a PEP. An important component of these studies will be defining the relevant treatment window in NHPs that corresponds with PEP in a clinical outbreak setting. It remains to be determined how a delayed ODV treatment would affect immune responses, specifically in comparison with development of natural immunity. However, even if ODV is only effective when given between 24 hours after EBOV exposure and before the onset of illness, it may have utility in combating EBOV outbreaks and for subjects with known exposures to EBOV, including accidents in the clinic or laboratories. This is important in the context of rapidly treating contacts, and contacts of contacts, of known EBOV exposures which can break the chain of transmission and better contain or stop an outbreak, especially in light of recent understandings of recrudescence events in survivors.

Small-molecule antivirals may have an advantage of crossing the blood-brain barrier as well as other immune privileged sites compared to large molecules (*59*, *60*). In this study, we consistently observed the presence of vRNA in all tissue types in the nontreatment and treatment failure macaques post-EBOV infection. In contrast, ODV treatment success macaques (*n* = 4 cyno and *n* = 5 rhesus) showed marked reduction or complete elimination of EBOV vRNA in immune-privileged tissues, including testes, brain, cervical spinal cord, and eyes (fig. S6). Our current finding is also consistent with SUDV infection, where all surviving cynomolgus monkeys treated with ODV showed no detectable vRNA in immune privileged tissues (*10*). No presence of vRNA was detected in the conjunctiva, the initial site of infection, in rhesus monkeys receiving ODV treatment. Only one of four ODV treatment success cynomolgus macaques showed a low level of viremia (1.7 × 10^4^ cp/g) compared to the ODV treatment failure and the vehicle control cynomolgus with vRNA levels between 1.3 × 10^5^ and 3.2 × 10^9^ cp/g. These findings support ODV’s ability to drive high systemic exposures to GS-441424 that extensively distribute into tissues and inhibit filovirus replication, presumed to include immune-privileged tissues.

Results from these preclinical NHP studies and our past work with SUDV (*10*) support further advancing ODV as a PEP for filovirus infections. Future testing of ODV treatment in NHPs beyond the early filovirus PEP should focus on evaluating the dose, the duration of treatment and delaying treatment until the onset of detectable viremia, and/or clinical signs of illness. Oral antivirals have several advantages over parenterally administered drugs, including potential to be easier to supply, store, distribute, and administer. These benefits of oral interventions promote the rapid and wide deployment as PEP particularly in a resource limited outbreak setting. Use of these more straightforward intervention approaches could also result in better acceptance and stronger support of medical countermeasures by populations in endemic areas and, together with the use of the recently licensed EBOV vaccines, to improve the response and control of future filovirus outbreaks.

## MATERIALS AND METHODS

### Study oversight

All study protocols described were approved by the University of Texas Medical Branch (UTMB) Institutional Animal Care and Use Committee (IACUC) which were compliant with UTMB Institutional Biosafety Committee guidelines under biosafety level 4 (BSL-4) containment. UTMB animal facilities used in this work are accredited by the Association for Assessment and Accreditation of Laboratory Animal Care International and adhere to principles specified in the eighth edition of the Guide for the Care and Use of Laboratory Animals, National Research Council.

### Virus and ODV

The EBOV Makona variant seed stock originated from serum from a fatal case during the 2014 outbreak in Guékédou, Guinea (*Ebola virus*/*H*.*sapiens*-*wt*/*GIN*/*2014*/*Makona*-*C07*, accession number KJ660347.2) and was passaged twice in authenticated Vero E6 cells obtained from American Type Culture Collection (ATCC; CRL-1586) (*61*, *62*). No mycoplasma or endotoxin could be detected (˂0.5 endotoxin units/ml). The 7 uracil (U) percentage of this EBOV seed stock is 100% (*61*), and the plaque-to-particle ratio is 25 to 1 as determined by Virocyt (Sartorius, Bohemia, NY). ODV drug substance used for the NHP study was prepared using a synthetic route previously described and purified by crystallization as form III (*63*).

### NHP challenge and treatment

A schematic of the ODV treatment studies in both cynomolgus and rhesus macaques is presented in fig. S7. In an initial study, six healthy captive bred cynomolgus macaques (*Macaca fascicularis*) (Worldwide Primates, Miami, FL) of Indonesian origin ~3 years of age and weighing ~ 2.6 to 3.3 kg were challenged by conjunctival exposure to 10,000 PFU of EBOV (Makona variant) as previously described (*14*, *64*). Briefly, 50 μl of challenge inoculum was delivered dropwise to the medial canthus of each eye for a total of 100 μl per animal. The eyelid was pulled out slightly to create a pocket for the inoculum and ensure secure administration of the challenge material. Assignment to the treatment group or the control group was determined before challenge by randomization by Excel. Five animals were treated by oral gavage with ODV (100 mg/kg; as a suspension in a 0.5% methyl cellulose in water vehicle) beginning 24 hours after EBOV exposure. These five animals received daily doses of ODV for 10 days (1 to 10 DPI). The EBOV-positive control animal was treated in parallel with 0.5% methyl cellulose in water. All ODV and vehicle treatments were performed under ketamine sedation.

To assess the pathogenic potential of EBOV (Makona variant) in rhesus monkeys (*Macaca mulatta*), four healthy captive bred macaques (Tulane Regional Primate Center, Covington, LA) of Chinese origin ~3 to 5 years of age and weighing ~3.4 to 6.4 kg were challenged by conjunctival exposure to 10,000 PFU of EBOV (Makona variant) as previously described (*14*, *64*).

After uniform lethality of the EBOV (Makona variant) administered by the conjunctival route in rhesus monkeys was confirmed, a second follow-up study with ODV was performed. Briefly, seven healthy captive bred rhesus macaques (Tulane Regional Primate Center, Covington, LA) of Chinese origin ~3 to 6 years of age and weighing ~3.9 to 7.1 kg were challenged by conjunctival exposure to 10,000 PFU of EBOV (Makona variant) as previously described (*14*, *64*). Assignment to the treatment group or control groups was determined before challenge by randomization by Excel. Five animals were treated by oral gavage with ODV beginning 24 hours after EBOV exposure. These five animals received daily doses of ODV through day 10 after EBOV challenge. One EBOV-positive control animal was treated in parallel with 0.5% methyl cellulose in water, while the other EBOV-positive control animal was not treated.

The duration of the study of all three NHP studies was 35 days. All 17 macaques were monitored daily and scored for disease progression with an internal EBOV humane endpoint scoring sheet approved by the UTMB ACUC. UTMB facilities used in this work are accredited by the Association for Assessment and Accreditation of Laboratory Animal Care International and adhere to principles specified in the eighth edition of the Guide for the Care and Use of Laboratory Animals, National Research Council. The scoring changes measured from baseline included posture and activity level, attitude and behavior, food intake, respiration, and disease manifestations, such as visible rash, hemorrhage, ecchymosis, or flushed skin. A score of ≥9 indicated that an animal met the criteria for euthanasia.

### Hematology and serum biochemistry

Total white blood cell counts, white blood cell differentials, red blood cell counts, platelet counts, hematocrit values, total hemoglobin concentrations, mean cell volumes, mean corpuscular volumes, and mean corpuscular hemoglobin concentrations were analyzed from blood collected in tubes containing EDTA using a Vetscan HM5 laser based hematologic analyzer (Zoetis). Serum samples were tested for concentrations of albumin, amylase, ALT, AST, alkaline phosphatase (ALP), BUN, calcium, CRE, CRP, GGT, glucose, total protein, and uric acid by using a Piccolo point-of-care analyzer and Biochemistry Panel Plus analyzer discs (Abaxis).

### RNA isolation from EBOV-infected macaques

Blood (100 μl) from K2-EDTA collection tubes was collected before centrifugation and was added to 600 μl of AVL viral lysis buffer with 6 μl of carrier RNA (QIAGEN, MD, USA, #52906) for RNA extraction. For tissues, approximately 100 mg was stored in 1 ml of RNAprotect (QIAGEN, #1018087) for at least 24 hours for stabilization. RNAprotect was completely removed, and tissues were homogenized in 600 μl of RLT buffer (QIAGEN, #74004) and 1% β-mercaptoethanol in a 2-ml cryovial using a TissueLyser II (QIAGEN, #85300) and 0.2-mm ceramic beads. The tissues sampled included axillary and inguinal lymph nodes, liver, spleen, kidney, adrenal gland, lung, brain, pancreas, urinary bladder, ovary or testis, conjunctiva, and eye. All blood samples were inactivated in AVL viral lysis buffer, and tissue samples were homogenized and inactivated in RLT buffer before removal from the BSL-4 laboratory. Subsequently, RNA was isolated from blood using the QIAamp vRNA kit (QIAGEN, #52906) and from tissues using the RNeasy minikit (QIAGEN, #74106) according to the manufacturer’s instructions supplied with each kit.

### Quantification of viral load

Primers and probe targeting the *VP30* gene of EBOV were used for RT-qPCR with the following probes: EBOV, 6-carboxyfluorescein (FAM)–5′ CCG TCA ATC AAG GAG CGC CTC 3′–6 carboxytetramethylrhodamine (TAMRA) (Life Technologies). vRNA was detected using the CFX96 detection system (Bio-Rad Laboratories, Hercules, CA) in one-step probe RT-qPCR kits (QIAGEN, #208356) with the following cycle conditions: 50°C for 10 min, 95°C for 10 s, and 40 cycles of 95°C for 10 s and 57°C for 30 s. Threshold cycle values representing viral genomes were analyzed with CFX Manager software, and the data are shown as genome equivalents per milliliter of blood or gram of tissue. To create the GEq standard, RNA from viral stocks was extracted, and the number of strain-specific genomes was calculated using Avogadro’s number and the molecular weight of each viral genome.

Virus titration was performed by plaque assay using Vero E6 cells (ATCC, CRL-1586) from all plasma and tissue samples as previously described (*61*, *62*). Briefly, increasing 10-fold dilutions of the samples were adsorbed to Vero E6 cell monolayers in duplicate wells (200 μl) and overlaid with 0.8% agarose in 1× Eagle’s minimum essentials medium (EMEM) with 5% fetal bovine serum and 1% penicillin-streptomycin. After a 6-day incubation at 37°C/5% CO_2_, neutral red stain was added, and plaques were counted after a 48-hour incubation. The limits of detection for this assay were 25 PFU/ml for plasma and 250 PFU/g for tissues.

### Plaque reduction neutralization test

Neutralization titers were calculated by determining the dilution of serum that reduced 50% of plaques (PRNT_50_). We incubated a standard 100 PFU amount of EBOV variant Makona with twofold serial dilutions of serum samples in EMEM for 1 hour. The virus-serum mixture was then used to inoculate Vero E6 cells (ATCC, CRL-1586) for 1 hour. Cells were overlaid with 2× MEM agar medium and incubated for 6 to 7 days, and plaques were counted after 24 hours of 5% neutral red staining.

### IgM and IgG ELISA

Immunosorbent MaxiSorp 96-well plates were coated overnight with 150 μl per well of recombinant EBOV GP (IBT Bioservices; catalog no. 0501-010; concentration, 0.08 μg/ml) in a sodium carbonate/bicarbonate solution (pH 9.6) as previously described (*65*). Antigen-adsorbed wells were subsequently blocked with 4% bovine serum antigen (BSA) in 1× phosphate-buffered saline (PBS) for at least 2 hours. Sera were initially diluted 1:100 and then twofold through 1:12,800 in ELISA diluent (1% BSA in 1× PBS and 0.2% Tween 20). After a 1-hour incubation, cells were washed four times with wash buffer (1× PBS with 0.2% Tween 20) and incubated for an hour with a dilution of horseradish peroxidase–conjugated anti-NHP IgM (1:5000) (Thermo Fisher Scientific, Rockford, IL) or IgG antibody (1:5000) (Fitzgerald Industries International, Acton, MA). SigmaFast *o*-phenylenediamine (OPD) substrate (Sigma-Aldrich, P9187) was added to the wells after four additional washes to develop the colorimetric reaction. The reaction was stopped with 2.5M sulfuric acid ~5 min after OPD addition, and absorbance values were measured at a wavelength of 490 nm on a Biotek Cytation 5. Absorbance values were determined by subtracting uncoated from antigen-coated wells at the corresponding serum dilution. IgM end-point titers were defined as the reciprocal of the last adjusted serum dilution with a value ≥ 0.30. IgG end-point titers were defined as the reciprocal of the last adjusted serum dilution with a value ≥ 0.20.

### NanoString sample preparation

Targeted transcriptomics was performed on blood samples from macaques as previously described (*66*). NHPV2_Immunology reporter and capture probe sets (NanoString Technologies) were hybridized with 3 μl of each RNA sample for ~24 hours at 65°C. The RNA:probe set complexes were subsequently loaded onto an nCounter microfluidic cartridge and assayed using a NanoString nCounter SPRINT Profiler. Samples with an image binding density greater than 2.0 or less than 0.20 were reanalyzed with 1 or 5 μl of RNA, respectively, to meet quality control criteria. All samples were included (no historical controls).

### Transcriptional analysis

nCounter .RCC files were imported into NanoString nSolver 4.0 software for analysis. To compensate for varying RNA inputs and reaction efficiency, an array of 10 housekeeping genes and spiked-in positive and negative controls were used to normalize the raw read counts as previously described (*65*, *67*). The array and the number of housekeeping mRNAs are selected by default within the NanoString nSolver Advanced Analysis module. As both sample input and reaction efficiency are expected to affect all probes uniformly, normalization for run-to-run and sample-to-sample variability is performed by dividing counts within a lane by the geometric mean of the reference/normalizer probes from the same lane (i.e., all probes/count levels within a lane are adjusted by the same factor). The ideal normalization genes are automatically determined by selecting those that minimize the pairwise variation statistic and are selected using the widely used geNorm algorithm as implemented in the Bioconductor package NormqPCR (*68*). The data were analyzed with NanoString nSolver Advanced Analysis 2.0 package for differential expression and to generate heatmap and cell type trend plots. Human annotations were added for each respective mRNA to perform immune cell profiling within nSolver. Normalized data (fold change and Benjamini-Hochberg adjusted *P* values) were exported as an .xlsx file and imported into GraphPad Prism version 9.3.1 to produce transcript heatmaps. These data are provided in the Supplementary Materials (data S1). For the heatmaps, samples from ODV-treated subjects for each NHP species were compared against control subjects at each selected time point (0, 4, 7, and 10 DPI). The topmost up-regulated and down-regulated transcripts (Benjamini-Hochberg adjusted *P* value < 0.05) are depicted and were sorted by the 10 DPI rhesus ODV-treated survivor group. For cell type trend plots, all samples (0, 4, 7, and 10 DPI) were compared from treated versus control subjects for each NHP species within nSolver. For the enrichment analysis, differentially expressed transcripts in treated versus control samples were evaluated using the Canonical Signaling Pathways module within Ingenuity Pathway Analysis (QIAGEN). The topmost up-regulated and down-regulated pathways were sorted by the 10 DPI rhesus ODV-treated survivor group.

### LegendPlex assays

Circulating levels of cytokine/chemokine- and coagulation-associated analytes were determined using LegendPlex bead-based multiplex technology (BioLegend) and NHP inflammation 13-plex (1:4 dilution) and human thrombosis 10-plex (1:100 dilution) kits. Plasma samples were processed in duplicate following the kit instructions and recommendations. Following bead staining and washing, at least 5000 bead events were collected on an Aurora cytometer (Cytek) using SpectroFlo software. The raw .fcs files were exported and analyzed with BioLegend’s cloud-based Qognit data analysis software. The concentration data based on known standards were exported to Microsoft Excel to calculate average fold change values (over a prechallenge baseline for each subject); the averages were then graphed in GraphPad Prism to generate the heatmap (version 10.0.1).

### Histopathology, IHC, and ISH

Necropsy was performed on all subjects. Tissue samples from major organs were collected for histopathological and IHC examination, immersion fixed in 10% neutral buffered formalin, and processed for histopathologic analysis as previously described (*61*, *62*). Tissue sections were deparaffinized and rehydrated through xylene and graded ethanols. Slides went through heat antigen retrieval in a steamer at 95°C for 20 min in Sigma-Aldrich citrate buffer (pH 6.0) (10×; Sigma-Aldrich, St. Louis, MO). The tissue sections were processed for IHC using the Thermo Autostainer 360 (Thermo Fisher Scientific, Kalamazoo, MI). Specific anti-EBOV VP40 immunoreactivity was detected using an anti-EBOV VP40 primary antibody (IBT Bioservices, #0301-010) at a 1:4000 dilution for 60 min. Secondary antibody used was biotinylated goat anti-rabbit IgG (Vector Laboratories, Burlingame, CA, #BA-1000) at 1:200 for 30 min followed by Vector Streptavidin Alkaline Phosphatase at a dilution of 1:200 for 15 min (Vector Laboratories, #SA-5100). Slides were developed with the ImmPact Red Substrate Kit (Vector Laboratories, #SK-5105) for 20 min and counterstained with hematoxylin for 30 s.

EBOV RNA ISH in formalin-fixed paraffin-embedded tissues was performed using the RNAscope 2.5 high-definition RED kit (Advanced Cell Diagnostics, Newark, CA) according to the manufacturer’s instructions. Twenty ZZ probe pairs targeting the genomic EBOV nucleoprotein gene were designed and synthesized by Advanced Cell Diagnostics (catalog no. 448581). After sectioning, deparaffinization with xylene and graded ethanol washes, and peroxidase blocking, the sections were heated in RNAscope Target Retrieval Reagent Buffer (Advanced Cell Diagnostics, catalog no. 322000) for 45 min and then air-dried overnight. The sections were then digested with protease IV (catalog no. 322336) at 40°C in the HybEZ oven (HybEZ, Advanced Cell Diagnostics, catalog no. 321711) for 30 min. Sections were exposed to ISH target probe and incubated at 40°C in the HybEZ oven for 2 hours. After rinsing, the signal was amplified using the manufacturer provided preamplifier and the amplifier conjugated to alkaline phosphatase, incubated with a red substrate–chromogen solution for 10 min, counterstained with hematoxylin, air-dried, and coverslipped.

## Supplementary Materials

### The PDF file includes:

Figs. S1 to S7 Tables S1 to S3 Legend for data s1
