# Supplementary material for: The oral drug obeldesivir protects nonhuman primates against lethal Ebola virus infection
Source: Sci Adv. 2025 Mar 14;11(11):eadw0659. doi: 10.1126/sciadv.adw0659 (PMC11908469; doi:10.1126/sciadv.adw0659)
Supplement: Supplementary file 1 — Figs. S1 to S7 Tables S1 to S3 Legend for data s1 [file sciadv.adw0659_sm.pdf]

Science Advances
AAAS

Supplementary Materials for

The oral drug obeldesivir protects nonhuman primates against lethal Ebola virus infection

Courtney Woolsey *et al.*

Corresponding author: Thomas W. Geisbert, twgeisbe@utmb.edu

Sci. Adv. **11**, eadw0659 (2025)
DOI: 10.1126/sciadv.adw0659

The PDF file includes:

Figs. S1 to S7
Tables S1 to S3
Legend for data s1

Other Supplementary Material for this manuscript includes the following:

Data S1

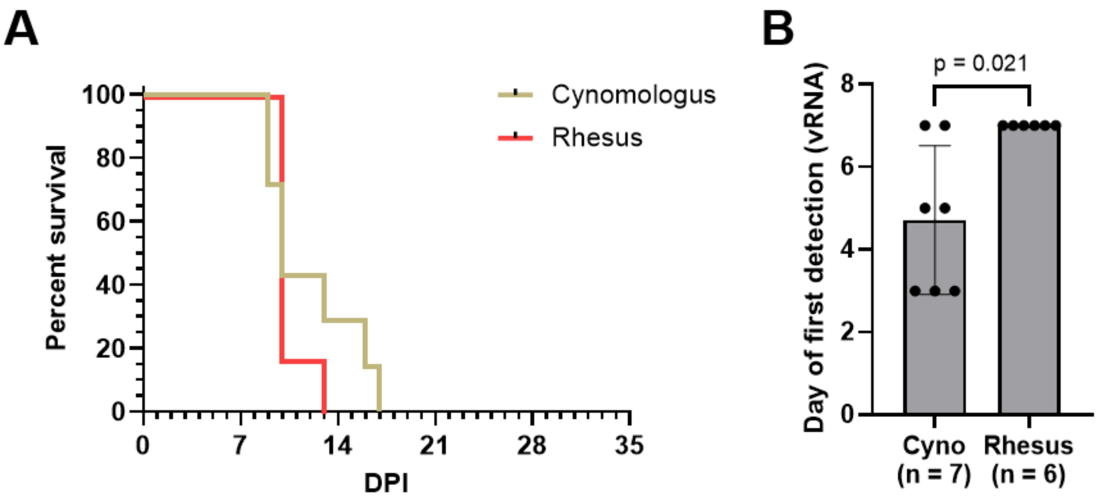

Figure S1: Comparison of survival and earliest detection of EBOV vRNA between untreated cynomolgus and rhesus macaques challenged with EBOV. (A) Kaplan-Meier survival curves for untreated EBOV-challenged (Makona variant) cynomolgus and rhesus macaques. Differences in curves were tested by the Mantel-Cox log rank test. **(B)** Comparison of the earliest detection of circulating EBOV vRNA in untreated cynomolgus and rhesus macaques as determined by RT-qPCR. Significance was determined using the non-parametric Mann-Whitney U-test.

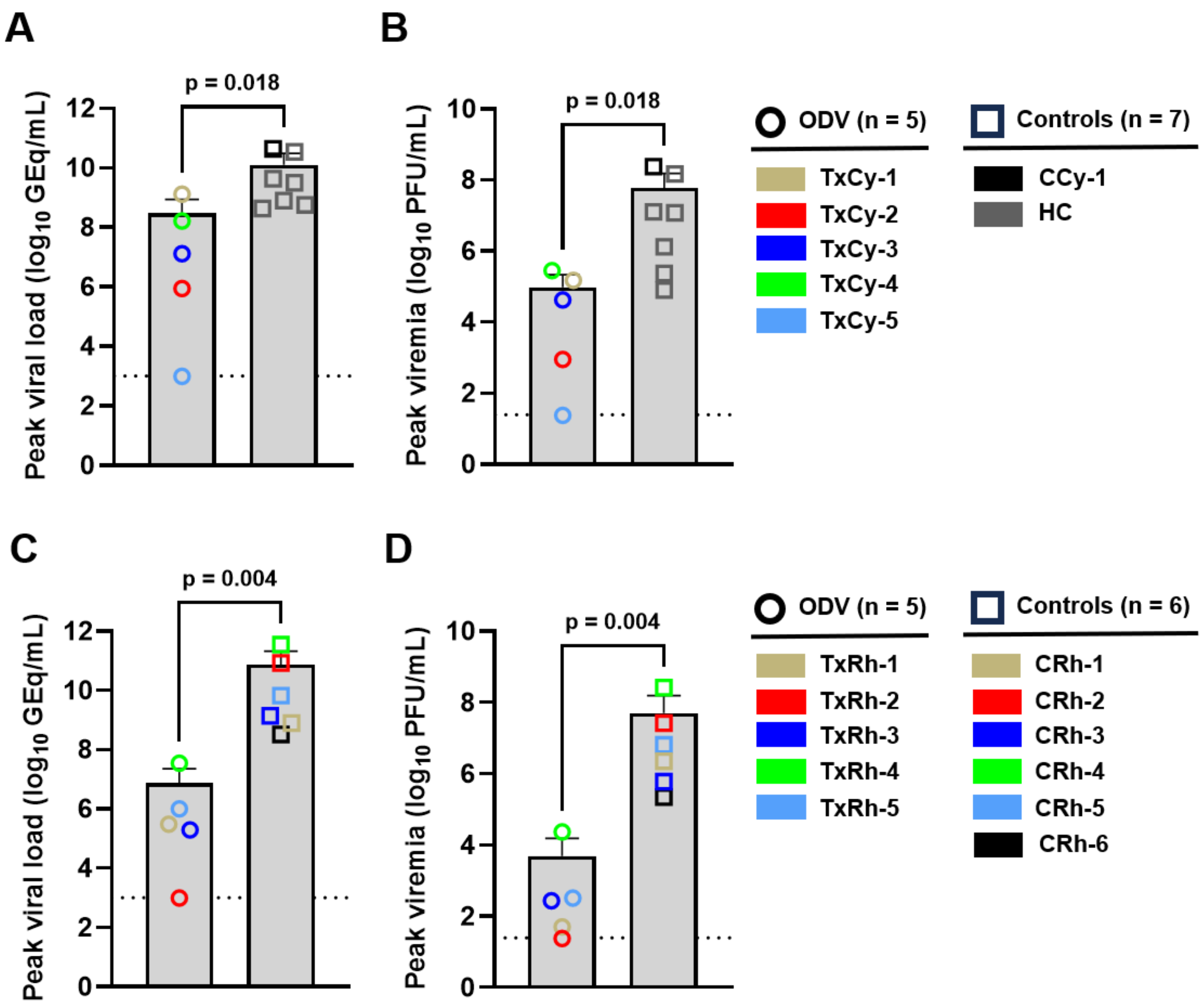

Figure S2: Reduction of viral burden in ODV-treated macaques. A comparison of the peak viral burden between ODV-treated and control animals was performed for both cynomolgus (**A, B**) and rhesus (**C,D**) macaques. The maximal values for viral load, as assessed by RT-qPCR of EBOV vRNA from whole blood (**A,C**), or circulating viremia, as assessed by plaque titration of plasma from each animal is plotted. For all panels, horizontal dashed lines denote the LLOQ for the assay (1000 GEq/mL for RT-qPCR, 25 PFU/mL for plaque titration). In animals without detectable vRNA or infectious virus, values just below the LLOQ (999 GEq/mL or 24 PFU/mL, respectively) were plotted. Significance was determined using the non-parametric Mann-Whitney U-test.

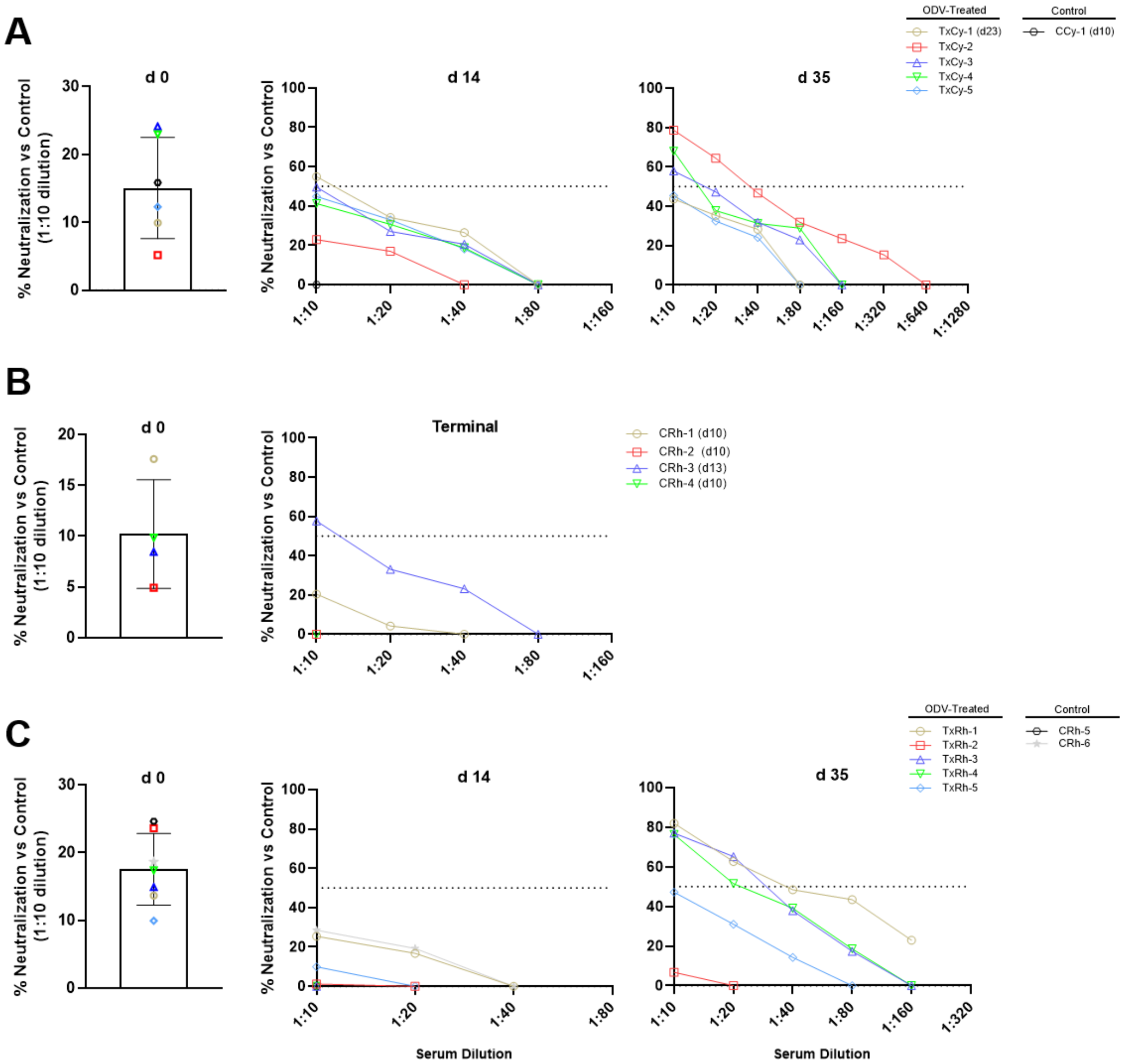

Figure S3: Serum antibody neutralization titers in cynomolgus and rhesus macaques challenged with EBOV. (A-C) Total anti-EBOV serum neutralization activity was determined for each animal by $PRNT_{50}$ at the indicated timepoints. **(A)** Serum antibody neutralization titers for cynomolgus macaques challenged with EBOV and treated with ODV. **(B)** Serum antibody neutralization titers for untreated rhesus macaques challenged with EBOV. **(C)** Serum neutralization titers for rhesus macaques challenged with EBOV and treated with ODV. For all panels, dashed lines indicate 50% neutralization compared to the virus control plate. Individual data points represent the mean of two technical replicates. For day of challenge (d 0) titers, bars indicate the arithmetic mean ± SD for the cohort. If the subject was euthanized prior to the pre-determine study endpoint (35 DPI), the terminal timepoint is indicated in parentheses next to the subject ID in the in-figure legend.

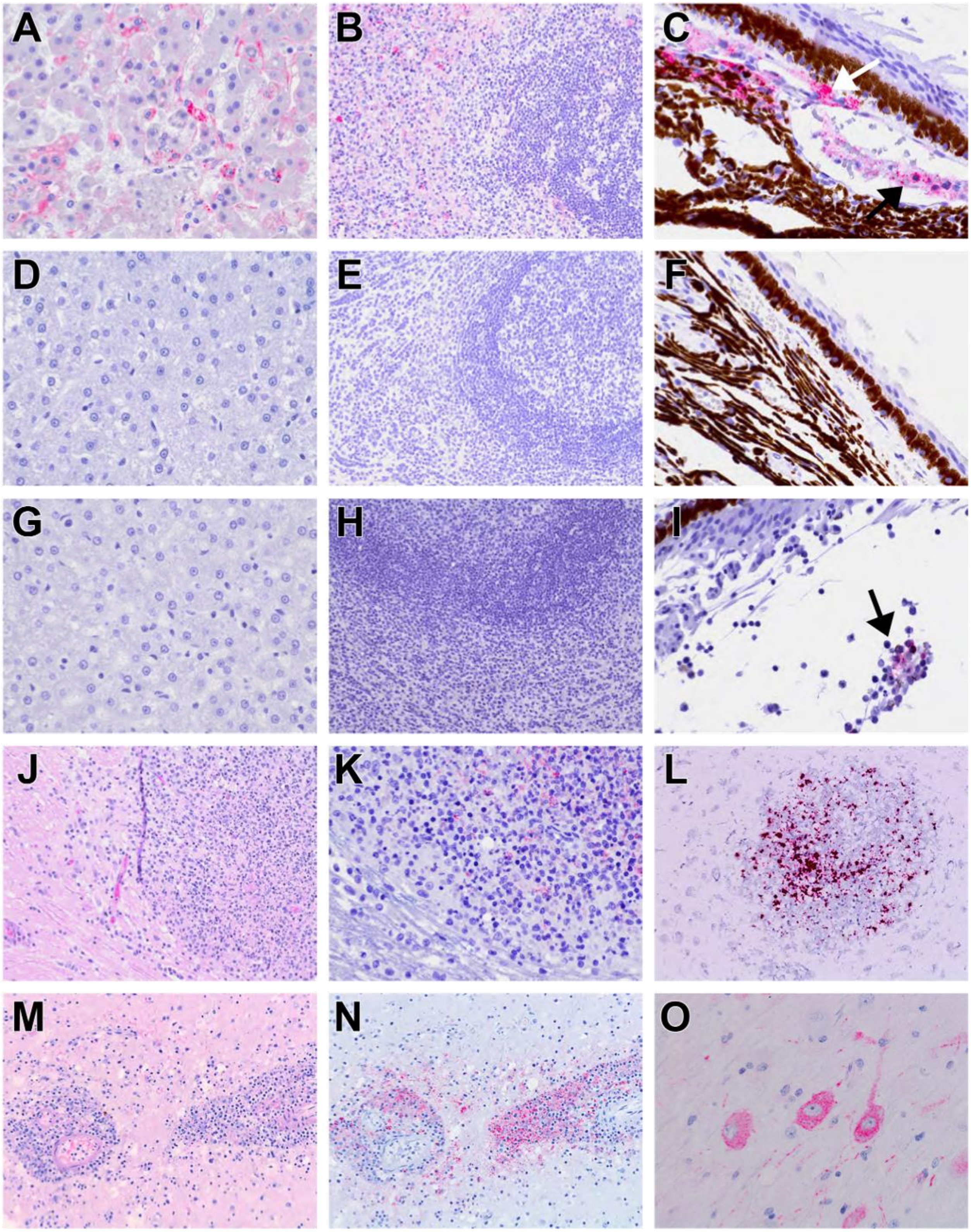

Figure S4: Pathology of ODV-treated and positive control cynomolgus monkeys. Representative hematoxylin and eosin (H&E: **J,M**) stained, immunohistochemistry (IHC: **A-I, K, N, O**) images for anti-EBOV VP40 antibody, and *in situ* hybridization (ISH: **L**) in cynomolgus monkeys from the in study positive control (CCy-1: **A-C**), ODV treatment survivor (TxCy-2: **D-F**), and ODV treatment with a delayed time to death, (TxCy-1:**G-O**), Images captured using 20x (**B,E,H,J,L-N**), and 40x (**A,C,D,F,G,I, K,O**) objectives (200x and 400x total magnification, respectively). Liver with immunolabeling (red) of Kupffer cells, hepatic sinusoidal lining cells, and rarely hepatocytes (**A**), no appreciable immunolabeling in the liver (**D, G**). Spleen with

immunolabeling of scattered individual mononuclear cells within the red and white pulp (**B**), no appreciable immunolabeling in the spleen (**E, H**). Choroid of the eye with immunolabeling of intravascular (**C**, black arrow) and extravascular (**C**, white arrow) mononuclear cells, no appreciable immunolabeling in the choroid (**F**). IHC positive mononuclear cells free within the vitreous chamber near the ora serrata (**I**, arrow). Brainstem with focal encephalitis (**J**). Higher magnification of the encephalitis with IHC positive (**K**) and ISH positive (**L**) inflammatory cells. Medial temporal lobe encephalitis with perivascular cuffing and vacuolar degeneration (**M**). Colocalized IHC positivity (**N**). IHC positive neuron like cells of the temporal lobe (**O**).

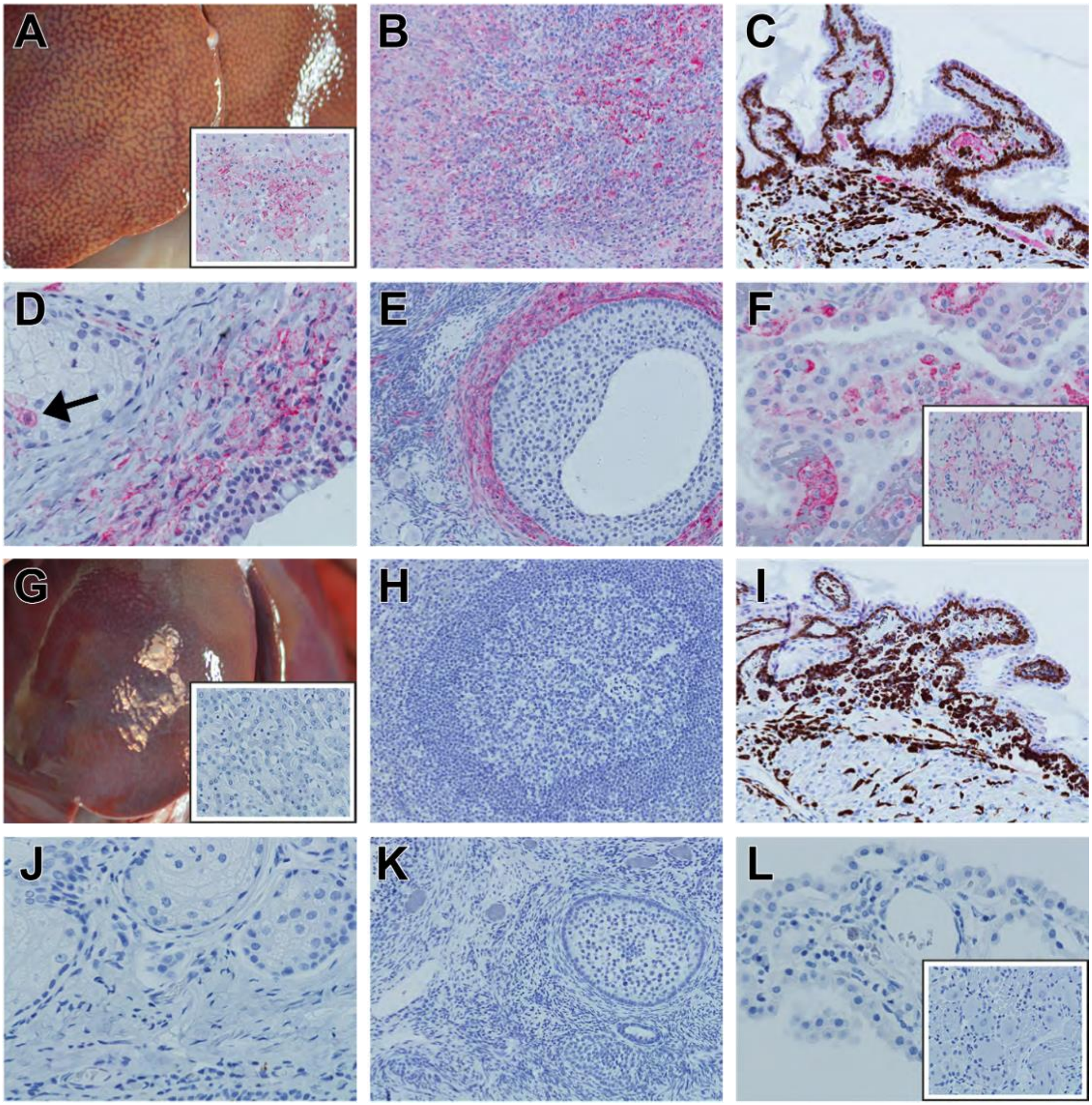

Figure S5: Pathology of ODV-treated and positive control rhesus monkeys. Representative gross images (CRh-3: **A**, TxRh-1: **G**) and immunohistochemistry (IHC: **A inset**, **B-F, F inset, G inset, H-L, L inset**) images for anti-EBOV VP40 antibody in rhesus monkeys from representative positive controls (CRh-4: **A inset, B-F, F inset**) and ODV treatment survivors (TxRh-5: **G inset, H-L, L inset**). Images captured using 20x (**B, C, E, H, I, K**) and 40x (**A inset, D, F, F inset, G inset, J, L, L inset**) objectives (200x and 400x total magnification, respectively). Reticulated pallor of the liver (**A**) and immunolabeling (red) of Kupffer cells, hepatic sinusoidal lining cells, and rarely hepatocytes (**A inset**). No appreciable gross lesions or immunolabeling in the liver (**G, G inset**). Spleen with immunolabeling of scattered individual mononuclear cells within the red and white pulp (**B**). No appreciable immunolabeling in the spleen (**H**). Ciliary body of the eye with immunolabeling of intravascular mononuclear cells (**C**). No appreciable immunolabeling in the ciliary body (**I**). IHC positive mononuclear cells free within dermis of the palpebre and sebocytes (**D, arrow**). No appreciable immunolabeling of the palpebre (**J).** Scattered interstitial cells and intensive immunolabeling of the theca in the ovary (**E**). No appreciable immunolabeling of the ovary (**K**). IHC positive intravascular mononuclear cells and endothelium of the choroid plexus (**F**) and positive interstitial cells, endothelium and satellite cells of the trigeminal ganglion (**F inset**). No appreciable immunolabeling of the choroid plexus (**L**) or trigeminal ganglion (**L inset**).

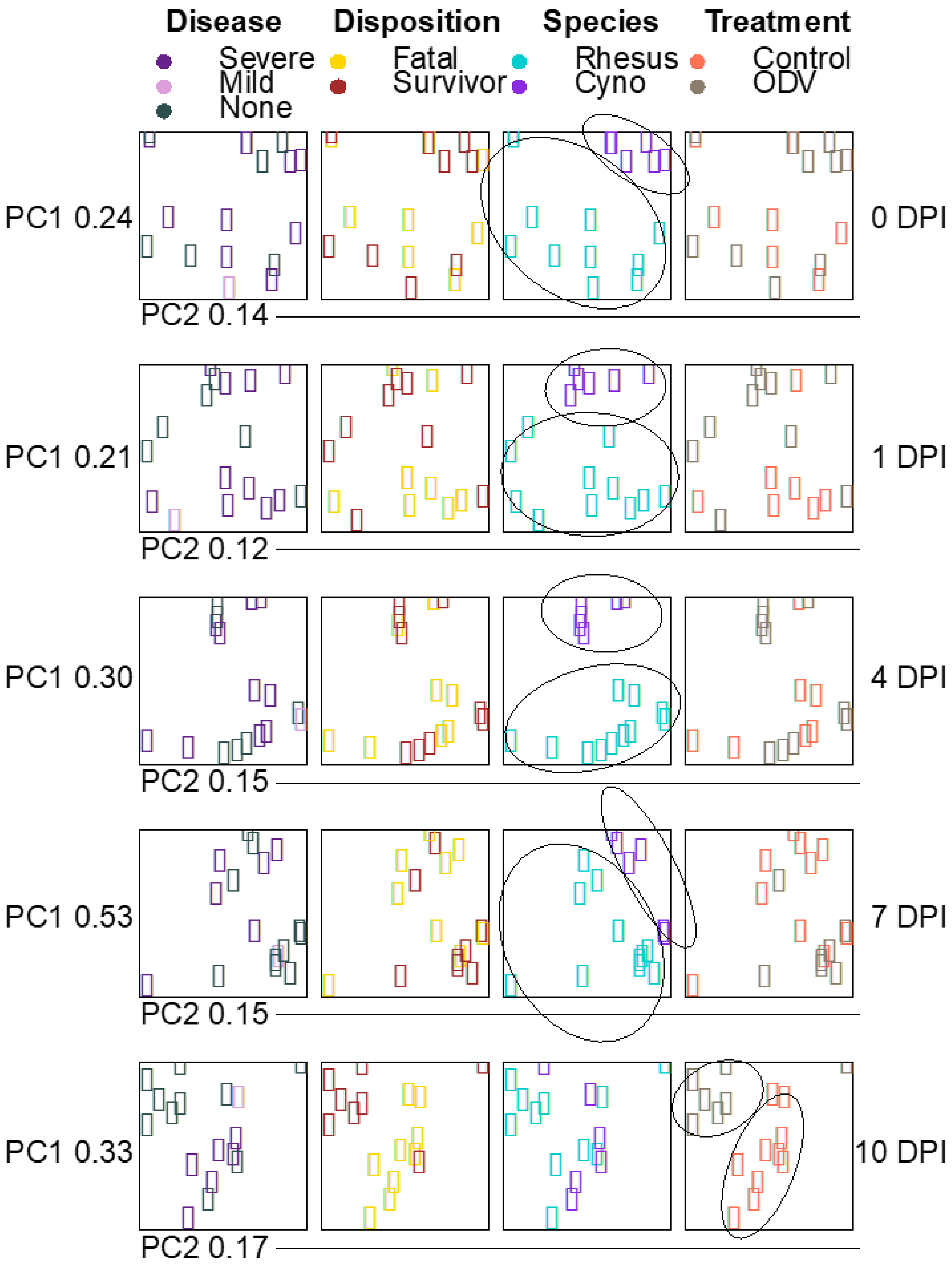

Figure S6: Dimensionality reduction of transcriptional changes in ODV-treated and control macaques. Principal component analysis (PCA) was applied at each selected DPI (0, 4, 7, 10 DPI) for dimensionality reduction to evaluate the contribution of four parameters—disease severity (none, mild, severe), final disposition (fatal, survivor), treatment (ODV, control), and NHP species—to the overall variability within the

dataset. Whole blood RNA samples were collected from EBOV-exposed cynomolgus (N=6; 5 treated, 1 in-study control) and rhesus macaques (N=11; 5 treated, 2 in-study controls, and 4 pilot study controls). Each dot represents an individual RNA sample; clustering indicates higher similarity between samples. PC1, principal component 1; PC2, principal component 2; DPI, days post infection; cyno, cynomolgus macaque; ODV, obeldesivir.

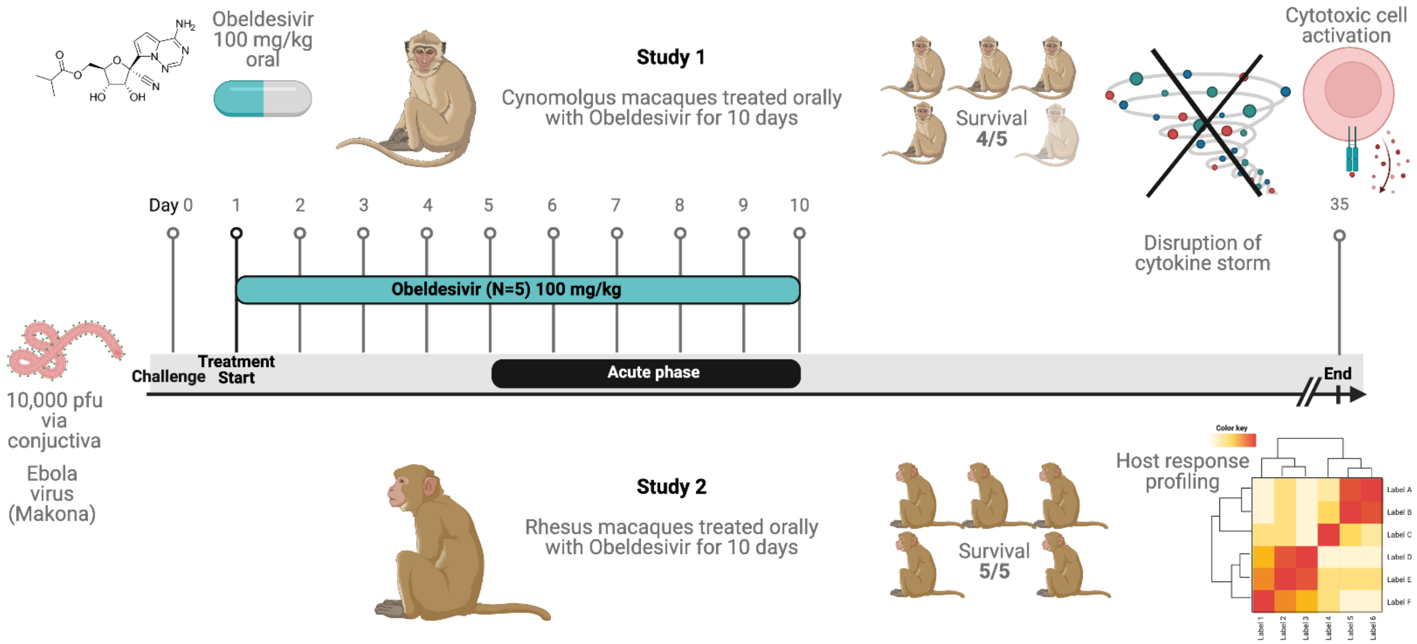

Figure S7: Schematic of experimental infection of cynomolgus and rhesus macaques with EBOV-Makona and rescue from lethal disease with obeldesivir.

Table S1. Clinical description and outcome of cynomolgus macaques following EBOV (Makona variant) conjunctival challenge with ODV treatment

NHP	Sex	Treatment	Clinical illness	Clinical pathology
TxCy-1	M	ODV	Fever (d14); decreased appetite (d14,20-23); anorexia (d15-19); petechial rash (d14-15); hunched posture (d18-23); depression (d18-23); weakness (d23); recumbency (d23); diarrhea (d17-19,22); dyspnea (d23); tremors (d21-23); convulsions (d23). Subject succumbed on d23.	Leukocytosis (d23); lymphopenia (d10); thrombocytopenia (d10,21,23); monocytopenia (d1); neutropenia (d4,7); eosinopenia (d4,7); basopenia (d4,7,23); monocytosis (d23); neutrophilia (d21,23); eosinophilia (d21); anemia (d21,23); hypoglycemia (d23); hypoalbuminemia (d21,23); CRE ↑ (d14); ALT ↑↑ (d14); AST ↑ (d21), ↑↑↑↑ (d14); ALP ↑ (d14); GGT ↑ (d14); CRP ↑ (d1,10,21,23), ↑↑↑ (d14).
TxCy-2	M	ODV	Decreased appetite (d10-11). Subject survived to study endpoint (d35).	Leukopenia (d10); lymphopenia (d10); thrombocytopenia (d10; monocytopenia (d10); neutropenia (d4,7,10,28,35); eosinopenia (d4,7,10,28,35); basopenia (d10); monocytosis (d14,28); hypoglycemia (d7); CRP ↑↑↑ (d10).
TxCy-3	M	ODV	Fever (d14); decreased appetite (d3,17-19); anorexia (d13-16); petechial rash (d14-16). Subject survived to study endpoint (d35).	Leukopenia (d10); lymphopenia (d10,14); thrombocytopenia (d14); monocytopenia (d14); neutropenia (d1,4,7,10,28,35); eosinopenia (d4); basopenia (d4,10,14); monocytosis (d1,21,28); neutrophilia (d14); ALT ↑ (d4,7,10,14,21,28); AST ↑↑↑ (d10); CRP ↑↑ (d14).
TxCy-4	F	ODV	Fever (d8-10); decreased appetite (d13-14); anorexia (d9, 11); petechial rash (d11-14); hunched posture (d18); depression (d15-18); Subject survived to study endpoint (d35).	Leukopenia (d10,28,35); lymphopenia (d7,10,14,21,28,35); thrombocytopenia (d10,14,28,35); monocytopenia (d1,21,28,35); neutropenia (d14,28,35); eosinopenia (d1,4,10,14,21,28,35); basopenia (d10,14,21,28,35); neutrophilia (d7); hypoproteinemia (d14); ALT ↑ (d10); AST ↑ (d10), ↑↑↑ (d14); ALP ↑ (d14); CRP ↑ (d1,7,14), ↑↑↑↑ (d10).
TxCy-5	F	ODV	None; Subject survived to study endpoint (d35).	Lymphopenia (d10,35); thrombocytopenia (d4); monocytopenia (d10,28,35); neutropenia (d10,14,21,28,35); eosinopenia (d4,7,10,14,21,18,35); basopenia (d4,10,21,28,35); monocytosis (d14,21); ALT ↑ (d14)
CCy-1	M	Control (Vehicle)	Fever (d8); hypothermia (d10); decreased appetite (d8); anorexia (d9-10); hunched posture (d9-10); depression (d10); weakness (d10); recumbency (d10); unresponsiveness (d10). Subject succumbed on d10.	Lymphopenia (d7,10); thrombocytopenia (d10); monocytopenia (d10); neutropenia (d4); eosinopenia (d4,10); basopenia (d4,10); hypoglycemia (d10); hypoalbuminemia (d10); hypoproteinemia (d10); hypoamylasemia (d10); BUN ↑ (d10); CRE ↑ (d10); ALT ↑ (d10); AST ↑↑↑ (d10); GGT ↑ (d10); CRP ↑↑↑ (d7), ↑↑↑↑ (d10).

Days after EBOV challenge are in parentheses. All reported findings are in comparison to baseline (day of challenge [d0]) values. Decreased appetite is defined as ≤ 65% of food consumed from the previous day. Anorexia is defined as no food consumed from the previous day. Fever is defined as a temperature more than 2.5 °F over baseline, or at least 1.5 °F over baseline and ≥ 103.5 °F. Hypothermia is defined as a temperature ≤3.5°F below baseline. Lymphocytopenia, monocytopenia, erythrocytopenia, thrombocytopenia, neutropenia, eosinopenia, and basopenia are defined by a ≥35% drop in numbers of lymphocytes, monocytes, erythrocytes, platelets, neutrophils, eosinophils, or basophils, respectively. Lymphocytosis, monocytosis, neutrophilia, eosinophila, and basophilia are defined by a 100% or greater increase in numbers of lymphocytes, monocytes, neutrophils, eosinophils, and basophils, respectively. Hyperglycemia is defined as a 100% or greater increase in levels of glucose. Hypoglycemia is defined by a ≥25% decrease in levels of glucose. Anemia is defined as a concurrent ≥25% decrease in erythrocyte count, Hct, and Hgb. Hypoalbuminemia is defined by a ≥25% decrease in levels of albumin. Hypoproteinemia is defined by a ≥25% decrease in levels of total protein. Hypoamylasemia is defined by a ≥25% decrease in levels of serum amylase. Hypocalcemia is defined by a ≥25% decrease in levels of serum calcium. Increases in ALT, AST, ALP, CRE, CRP, Hct, and Hgb were graded on the following scale: ↑ = 1-5 fold, ↑↑ = >5-10 fold, ↑↑↑ = >10-20 fold, ↑↑↑↑ = >20-fold, ↓ = ≥50% decrease. (BUN) blood urea nitrogen, (ALT) alanine aminotransferase, (AST) aspartate aminotransferase, (ALP) alkaline phosphatase, (CRE) Creatinine, (CRP) C-reactive protein, (Hct) hematocrit, (Hgb) hemoglobin.

Table S2. Clinical description and outcome of rhesus macaques following EBOV (Makona variant) conjunctival challenge

NHP	Sex	Treatment	Clinical illness	Clinical pathology
CRh-1	M	None	Fever (d7); hypothermia (d10); decreased appetite (d9); anorexia (d10); petechial rash (d9-10); hunched posture (d9-10); depression (d10); rectal bleeding (d10). Subject succumbed on d10.	Leukopenia (d10); lymphopenia (d7,10); thrombocytopenia (d10); neutropenia (d4,10); eosinopenia (d10); basopenia (d10); monocytosis (d4,7,10); anemia (d21,23); hypoalbuminemia (d10); BUN ↑↑ (d10); CRE ↑↑↑ (d10); ALT ↑↑↑ (d10); AST ↑↑↑↑ (d10); ALP ↑ (d10); GGT ↑ (d10); CRP ↑↑↑↑ (d7,10)
CRh-2	M	None	Fever (d7); hypothermia (d10); anorexia (d9-10); petechial rash (d10); hunched posture (d10); weakness (d10); recumbency (d10); rectal bleeding (d10). Subject succumbed on d10.	Lymphopenia (d10); neutropenia (d4,10); eosinopenia (d10); basopenia (d10); lymphocytosis (d10); monocytosis (d4,7,10); hypoalbuminemia (d10); hyperamylasemia (d10); BUN ↑↑ (d10); CRE ↑↑↑ (d10); ALT ↑↑↑↑ (d10); AST ↑↑↑↑ (d10); ALP ↑ (d10); GGT ↑↑ (d10); CRP ↑↑↑ (d7), ↑↑↑↑ (d10).
CRh-3	F	None	Fever (d10); decreased appetite (d1-2); anorexia (d11-13); petechial rash (d12-13); hunched posture (d12-13); depression (d13); recumbency (d13). Subject succumbed on d13.	Lymphopenia (d10,13); thrombocytopenia (d13); neutropenia (d4); eosinopenia (d4,13); basopenia (d13); monocytosis (d10); neutrophilia (d10); eosinophilia (d10); hypoamylasemia (d13); BUN ↑ (d13); CRE ↑ (d13); ALT ↑↑↑ (d13); AST ↑↑↑↑ (d13); ALP ↑ (d13); GGT ↑ (d13); CRP ↑↑↑↑ (d10,13).
CRh-4	F	None	Fever (d7); hypothermia (d10); anorexia (d9-10); petechial rash (d10); hunched posture (d10); depression (d10); weakness (d10); recumbency (d10). Subject succumbed on d10.	Lymphopenia (d7); thrombocytopenia (d10); neutropenia (d4,10); eosinopenia (d4); basopenia (d10); monocytosis (d4,10); neutrophilia (d7); eosinophilia (d7); hypoglycemia (d10); hypoalbuminemia (d10); BUN ↑ (d10); CRE ↑↑ (d10); ALT ↑↑↑↑ (d10); AST ↑↑↑↑ (d10); ALP ↑ (d10); GGT ↑↑ (d10); CRP ↑↑↑ (d7), ↑↑↑↑ (d10).

Days after EBOV challenge are in parentheses. All reported findings are in comparison to baseline (day of challenge [d0]) values. Decreased appetite is defined as ≤ 65% of food consumed from the previous day. Anorexia is defined as no food consumed from the previous day. Fever is defined as a temperature more than 2.5 °F over baseline, or at least 1.5 °F over baseline and ≥ 103.5 °F. Hypothermia is defined as a temperature ≤3.5°F below baseline. Lymphocytopenia, monocytopenia, erythrocytopenia, thrombocytopenia, neutropenia, eosinopenia, and basopenia are defined by a ≥35% drop in numbers of lymphocytes, monocytes, erythrocytes, platelets, neutrophils, eosinophils, or basophils, respectively. Lymphocytosis, monocytosis, neutrophilia, eosinophila, and basophilia are defined by a 100% or greater increase in numbers of lymphocytes, monocytes, neutrophils, eosinophils, and basophils, respectively. Hyperglycemia is defined as a 100% or greater increase in levels of glucose. Hypoglycemia is defined by a ≥25% decrease in levels of glucose. Anemia is defined as a concurrent ≥25% decrease in erythrocyte count, Hct, and Hgb. Hypoalbuminemia is defined by a ≥25% decrease in levels of albumin. Hypoproteinemia is defined by a ≥25% decrease in levels of total protein. Hypoamylasemia is defined by a ≥25% decrease in levels of serum amylase. Hypocalcemia is defined by a ≥25% decrease in levels of serum calcium. Increases in ALT, AST, ALP, CRE, CRP, Hct, and Hgb were graded on the following scale: ↑ = 1-5 fold, ↑↑ = >5-10 fold, ↑↑↑ = >10-20 fold, ↑↑↑↑ = >20-fold, ↓ = ≥50% decrease. (BUN) blood urea nitrogen, (ALT) alanine aminotransferase, (AST) aspartate aminotransferase, (ALP) alkaline phosphatase, (CRE) Creatinine, (CRP) C-reactive protein, (Hct) hematocrit, (Hgb) hemoglobin.

Table S3. Clinical description and outcome of rhesus macaques following EBOV (Makona variant) conjunctival challenge with ODV treatment

NHP	Sex	Treatment	Clinical illness	Clinical pathology
TxRh-1	M	ODV	Fever (d7-10); decreased appetite (d10); Subject survived to study endpoint (d35).	Leukocytosis (d7,14); lymphopenia (d7); lymphocytolysis (d1,14); monocytopenia (d4,7,10,14,21), neutrophilia (d4,7,10,21,28); neutropenia (d35); eosinophilia (d1,4,7,10,14,21,28); basophilia (d1,4,7,14,21,28); AST ↑ (d10); CRP ↑ (d1), ↑↑ (d7,10)
TxRh-2	M	ODV	Mild weakness (d22-27); Subject survived to study endpoint (d35).	Leukopenia (d28); lymphopenia (d14); monocytopenia (d7,10,14,21,28); neutrophilia (d4,7,10,21); neutropenia (d35); eosinophilia (d4,7); ALT ↑ (d10)
TxRh-3	M	ODV	Fever (d8-10); Subject survived to study endpoint (d35).	Lymphopenia (d10); monocytopenia (d7,14,35); neutrophilia (d7,10,14,21,28); eosinopenia (d10,14,35); basopenia (d10); hyperamylasemia (d10); AST ↑ (d10); CRP ↑↑↑↑ (d10)
TxRh-4	M	ODV	Fever (d8-10); decreased appetite (d9,11,14,15); anorexia (d10); Subject survived to study endpoint (d35).	Leukopenia (d10); lymphopenia (d7,10: thrombocytopenia (d10,14); monocytosis (d1,7,10.14,21,28,35); neutropenia (d1,10,14,21,35); neutrophilia (d7); eosinopenia (d1); eosinophilia (d7); basopenia (d1,10,14); hypoamylasemia (d10,21); AST ↑ (d10,14); CRP ↑ (d7,14), ↑↑↑↑ (d10)
TxRh-5	F	ODV	Fever (d9-10); Subject survived to study endpoint (d35).	Lymphopenia (d7,10); monocytopenia (d4,7,10,21); neutrophilia (d1,4,7,10,14,21,28); neutropenia (d35); eosinophilia (d28); hypoamylasemia (d10); AST ↑ (d10); ALP ↑ (d14); CRP ↑ (d10)
CRh-5	M	None	Fever (d7); hypothermia (d10); anorexia (d8-10); hunched posture (d8-10); depression (d10); petechial rash (d8-10); recumbency (d10); diarrhea (d10); Subject succumbed on d10.	Leukopenia (d10); lymphopenia (d7,10); thrombocytopenia (d10); monocytopenia (d1,7,10); neutrophilia (d1,7,10); eosinophilia (d10: eosinopenia (d10); basopenia (d10); hypoalbuminemia (d10); hypoglycemia (d7,10); hypoamylasemia (d7,10); BUN ↑ (d10); ALT ↑↑ (d10); AST ↑ (d7), ↑↑↑↑ (d10); GGT ↑ (d10); CRP ↑↑↑ (d7), ↑↑↑↑ (d10)
CRh-6	M	Control (Vehicle)	Fever (d6-9); decreased appetite (d9); anorexia (d10); depression (d10); petechial rash (d8-10); hunched posture (d10); recumbency (d10); bleeding from nares (d10); Subject succumbed on d10.	Lymphopenia (d7,10); thrombocytopenia (d7,10); monocytopenia (d1); monocytosis (d7,10); neutrophilia (d1,4,7); eosinophilia (d7); basopenia (d10); hypoamylasemia (d7,10); ALT ↑ (d10); AST ↑↑↑↑ (d10); ALP ↑ (d10); CRP ↑↑↑↑ (d7,10)

Days after EBOV challenge are in parentheses. All reported findings are in comparison to baseline (day of challenge [d0]) values. Decreased appetite is defined as ≤ 65% of food consumed from the previous day. Anorexia is defined as no food consumed from the previous day. Fever is defined as a temperature more than 2.5 °F over baseline, or at least 1.5 °F over baseline and ≥ 103.5 °F. Hypothermia is defined as a temperature ≤3.5°F below baseline. Lymphocytopenia, monocytopenia, erythrocytopenia, thrombocytopenia, neutropenia, eosinopenia, and basopenia are defined by a ≥35% drop in numbers of lymphocytes, monocytes, erythrocytes, platelets, neutrophils, eosinophils, or basophils, respectively. Lymphocytosis, monocytosis, neutrophilia, eosinophila, and basophilia are defined by a 100% or greater increase in numbers of lymphocytes, monocytes, neutrophils, eosinophils, and basophils, respectively. Hyperglycemia is defined as a 100% or greater increase in levels of glucose. Hypoglycemia is defined by a ≥25% decrease in levels of glucose. Anemia is defined as a concurrent ≥25% decrease in erythrocyte count, Hct, and Hgb. Hypoalbuminemia is defined by a ≥25% decrease in levels of albumin. Hypoproteinemia is defined by a ≥25% decrease in levels of total protein. Hypoamylasemia is defined by a ≥25% decrease in levels of serum amylase. Hypocalcemia is defined by a ≥25% decrease in levels of serum calcium. Increases in ALT, AST, ALP, CRE, CRP, Hct, and Hgb were graded on the following scale: ↑ = 1-5 fold, ↑↑ = >5-10 fold, ↑↑↑ = >10-20 fold, ↑↑↑↑ = >20-fold, ↓ = ≥50% decrease. (BUN) blood urea nitrogen, (ALT) alanine aminotransferase, (AST) aspartate aminotransferase, (ALP) alkaline phosphatase, (CRE) Creatinine, (CRP) C-reactive protein, (Hct) hematocrit, (Hgb) hemoglobin.

Data S1. $-\log_{10}$(P values) and $\log_2$(fold change) data from Nanostring targeted transcriptome profiling.
